# The effect of maternal decisional authority on children's vaccination in East Asia

**DOI:** 10.1371/journal.pone.0200333

**Published:** 2018-07-12

**Authors:** Minsoo Jung

**Affiliations:** Department of Health Science, Dongduk Women's University, Seoul, South Korea; TNO, NETHERLANDS

## Abstract

Even though they are important determinants for increasing vaccination rates in advanced and developing nations alike, maternal capacity and decisional authority have not been fully elucidated in diverse countries and cultural spheres. This study examined the effects of South Korean, Chinese, and Japanese mothers’ health literacy, self-efficacy, mass media use, and decisional authority on their children’s vaccination after adjustment for their socioeconomic statuses. Computer-assisted web interviews were conducted with married women in their 20s-40s of South Korean, Chinese, or Japanese nationality (n = 1,571). Dependent variables were generated for the following four vaccinations: BCG, diphtheria+pertussis+tetanus (DPT), poliomyelitis (polio), and measles. For statistical processing, cases where all four types of vaccines had been recorded were scored as 1 and other cases were processed as 0. According to the results of the pooled model, we found that for East Asian mothers, decisional authority, self-efficacy, and health literacy all increased the likelihood that they would vaccinate their children. Furthermore, women who searched for health information through media such as the radio were more likely to vaccinate their children. However, when elaborate analyses were conducted by country, there were considerable differences in those characteristics by country. Therefore, this study showed that it is necessary to establish locally tailored strategies in order to raise vaccination rates in the Global Vaccine Action Plan. This study also showed that social contexts must be taken into consideration in order to raise vaccination rates.

## Introduction

Although the prevalence of vaccine-preventable diseases has decreased steadily, 1/3 of countries worldwide have yet to reach an immunization rate of 90%, which is the goal of the World Health Organization’s (WHO) Global Vaccine Action Plan (GVAP) [1]. As of 2013, over 20 million children aged less than one year still failed to receive not only diphtheria-pertussis-tetanus (DPT) vaccines but also one-time immunizations such as against measles [2].

Parents’ socioeconomic characteristics are associated with the failure to receive vaccination or with incomplete vaccination (for example, the oral polio vaccine against poliomyelitis must be received four times up to the age of five). Not only parents’ ages, education levels, poverty, religions, children’s gender, and distance from medical facilities [3–12] but also trust in medical staff and social trust have been reported to be associated with immunization [13–15]. This phenomenon is more frequently observed in particular among mothers who have received insufficient education and lack capacity. Vaccination rates can be increased to create safe societies only when these mothers’ children become the targets of supplementary immunization activities.

However, unlike in the past, when physical access to immunization was the main issue, diverse socio-contextual variables including parental characteristics, especially mothers’ capacity (health literacy, self-efficacy, and decisional authority) and mass media use, have become important in recent years [15–18]. In particular, in both advanced and developing nations, where the associations between parents’ socioeconomic characteristics and children’s immunization are low, negative attitudes such as parents’ vaccine reluctance or delays have emerged as new problems [19]. This presumably is affected also by cultural characteristics that either are patriarchal or restrict mothers’ autonomy.

Partial or incomplete vaccination is strongly associated with poverty, illiteracy, and mothers’ youth [20–22]. Consequently, the roles of mothers in children’s health have been continued to be elucidated in various countries and regions. However, even though they are important determinants for increasing vaccination rates in advanced and developing nations alike, maternal capacity and decisional authority have not been fully elucidated in diverse countries and cultural spheres [20–23]. In particular, there has been little research on the effects of maternal capacity on children’s immunization in East Asia, where patriarchal and Confucian characteristics remain. In this study, we examined the effects of South Korean, Chinese, and Japanese mothers’ health literacy, self-efficacy, mass media use, and decisional authority on vaccinating their children after we adjusted for the women’s socioeconomic statuses.

## Methods

### Study sample

We drew our data from a survey of respondents drawn from a nationally representative online sample of women in East Asia who participated in the Hankook Research Master Sample Panel (Korean) and Lightspeed Global Market Insite, Inc Sample Panel (Chinese and Japanese) by the request of the principal investigator of this study (S1–S4 Files). We conducted computer-assisted web interviews with married women in their 20s-40s of South Korean, Chinese, or Japanese nationality. Although the total sample size was 2,134 individuals, we only extracted 1,571 who had underage children and who answered questions on vaccinating their children, and used them in the final analysis (574 Korean; 566 Chinese; 431 Japanese). Respondents received a nominal cash incentive (USD $2.00) to participate when they completed the surveys, and the final response rate was 87%. We excluded missing values for the survey questions for key analytical variables using the pairwise method.

### Study design

This was a cross-sectional study to examine the associations between women’s decisional authority and their vaccinating their children, with mass media use as a moderator.

### Measures

#### Dependent variables

We measured the dependent variable for this study, mothers’ vaccinating children, by asking “Now think about vaccinating your child/children. Have you vaccinated one or all of your children?” The responses were yes or no. We generated four binary variables for the following four vaccinations: BCG, (DPT), polio, and measles. For statistical processing, we recorded cases where children had received all four vaccines as 1 and processed all other cases as 0.

#### Independent variables. Maternal capacity

We assessed maternal capacity based on their decision-making authority regarding the household finances. Use of this dimension has been validated in previous studies on women’ empowerment [24]. For this study, we measured the women’s decisional authority using the following five questions: “Who usually makes decisions on how to spend your income?” “Who usually makes decisions on how to spend your spouse’s income?” “Who makes decisions on your use of medical services?” “Who usually purchases objects in your house?” “Who usually makes decisions on visits to family members or relatives?” There were three possible responses: (1) partner decides, (2) partner and woman decide jointly, and (3) woman decides. We conducted confirmatory factor analysis (CFA) with the five questions to multi-dimensionally assess the women’s capacity [24]. The factors we used to construct the capacity index presented eigenvalues greater than 1 and factor loadings greater than 0.40. The single factor decisional authority accounted for 48.7% of the total variance (Cronbach’s alpha = 0.770).

We considered media use as a moderator in the model in accordance with the literature [15, 25] and measured the respondents’ health-related media use using the following five questions: “Did you seek health information through the television, radio, newspapers, books, or Internet over the past week?” These items had three responses: (1) not at all, (2) 1–2 times, and (3) more than three times.

We also considered self-efficacy and health literacy as covariates between socioeconomic characteristics and vaccination behavior in accordance with the literature [16]. We measured the women’s self-efficacy using the following five questions: “I am confident,” “I can perform my duties as planned,” “Even when something goes wrong at the beginning, I try to finish a task,” “I can accomplish important goals when I set them,” and “I start right away when there is something to do,” and there were five responses: (1) I strongly disagree; (2) I disagree; (3) I neither agree nor disagree; (4) I agree; (5) I agree strongly. Based on the CFA results, a single factor, self-efficacy, explained 44.2% of the total variance (Cronbach’s alpha = 0.708). We measured the women’s health literacy using the following five questions: “Can you understand and complete forms provided by hospitals (examples: surgery consent forms, diagnosis and examination guidelines, patient medication instructions)?” “Can you understand the contents of medical charts for patient management and write in the necessary contents?” “Can you understand printed materials that have medical information provided by physicians?” “Do you know how to make reservations for diagnoses and examinations?” “Can you ask physicians questions in order to understand unfamiliar health information?” There were three response options: (1) “I often have difficulty”; (2) “I sometimes have difficulty”; (3) “I have no difficulty.” The CFA results showed that self-efficacy explained 55.2% of the total variance (Cronbach’s alpha = 0.808).

#### Potential confounders

It has been previously reported that maternal health is affected by demographic characteristics [26, 27]. In accordance with these reports, we used mother’s age, educational attainment, and monthly household income as the baseline independent variables. We grouped the participants into the following age categories, 20–25, 26–30, 31–35, and 36–40 years, and education attainment into high school/associate’s degree or below, bachelor’s degree, or graduate school or higher. We divided household income into below $26,999, $27,000–$35,499, $35,500–$44,499, $44,500–$52,999, $53,000–$61,999, $62,000–$70,999, $71,000 or more in US dollars.

### Statistical analyses

First, we described the general characteristics of the sample for each country in East Asia, including assessing media use and vaccination rates. Second, we conducted hierarchical multivariable regression analysis to examine the relationships between the women’s decisional authority factors and their vaccinating their children after we adjusted for potential confounders. Finally, we assessed the associations between women’s decisional authority and vaccination stratified by nationality (South Korea, China, and Japan). We performed all statistical analyses using STATA v. 14.0 (STATA, College Station, TX, USA).

### Ethics statement

Approval for the study was granted by the Korea National Institute for Bioethics Policy Institutional Review Board (November 25, 2016; P01-201611-21-009). All participants gave written informed consent to participate. During the investigation process, we collected no information that could distinguish individual participants.

## Results

### Descriptive statistics of the sample

Table 1 shows South Korean, Chinese, and Japanese women’s national vaccination rates with respect to their underage children, socioeconomic characteristics, and media use. First, immunization rates against the four diseases were the highest for South Korean mothers, with rates for tuberculosis (TB) and DPT of 98.4% and 98.4%, respectively. On the contrary, in the case of Japanese mothers, immunization rates were only 79.4% and 84.5% for measles, mumps, and rubella and polio, respectively. Next, the respondents’ education levels were the highest for Chinese mothers, with a rate of 93.9% for high education (college/university graduates and above) compared with only 69.8% in Japan. However, the monthly household income was US$71,000 or above for 45.0% of the Japanese respondents, making them wealthier than their South Korean and Chinese peers. Finally, the results for media use showed that women from all three countries primarily located health information from the Internet and the television. By nationality, South Korean mothers used the Internet, Chinese mothers read newspapers or listened to the radio, and Japanese mothers used the Internet.

**Table 1:** Descriptive statistics of the sample (n = 1571).

		Countries			X^2^	P-value
		S. Korea	China	Japan		
Vaccination	BCG Yes	565 (98.4%)	476 (84.1%)	372 (86.3%)	73.383	0.000
	No	9 (1.6%)	90 (15.9%)	59 (13.7%)		
	POLIO Yes	556 (96.9%)	503 (88.9%)	364 (84.5%)	47.455	0.000
	No	18 (3.1%)	63 (11.1%)	67 (15.5%)		
	DPT Yes	565 (98.4%)	515 (91.0%)	376 (87.2%)	49.185	0.000
	No	9 (1.6%)	51 (9.0%)	55 (12.8%)		
	Measles Yes	534 (93.0%)	518 (91.5%)	342 (79.4%)	52.957	0.000
	No	40 (7.0%)	48 (8.5%)	89 (20.6%)		
Education	High school or below	89 (15.5%)	35 (6.2%)	130 (30.2%)	127.728	0.000
	Bachelor’s degree	437 (76.1%)	516 (91.2%)	275 (63.8%)		
	Graduate school or higher	48 (8.4%)	15 (2.7%)	26 (6.0%)		
Monthly	US$26,999 or below	80 (13.9%)	126 (22.3%)	16 (3.7%)	183.975	0.000
Income	US$27,000-US$35,499	93 (16.2%)	79 (14.0%)	32 (7.4%)		
	US$35,500-US$44,499	100 (17.4%)	82 (14.5%)	36 (8.4%)		
	US$44,500-US$52,999	86 (15.0%)	74 (13.1%)	47 (10.9%)		
	US$53,000-US$61,999	63 (11.0%)	53 (9.4%)	53 (12.3%)		
	US$62,000-US$70,999	47 (8.2%)	41 (7.2%)	53 (12.3%)		
	US$71,000 or more	105 (18.3%)	111 (19.6%)	194 (45.0%)		
Media Use	Television Not at all	262 (45.6%)	150 (26.5%)	319 (74.0%)	274.284	0.000
	1–2 times	253 (44.1%)	247 (43.6%))	89 (20.6%))		
	More than 3 times	59 (10.3%)	169 (29.9%)	23 (5.3%)		
	Radio Not at all	513 (89.4%)	307 (54.2%)	419 (97.2%)	335.592	0.000
	1–2 times	54 (9.4%)	190 (33.6%)	7 (1.6%)		
	More than 3 times	7 (1.2%)	69 (12.2%)	5 (1.2%)		
	Newspaper Not at all	502 (87.5%)	223 (39.4%)	401 (93.0%)	467.796	0.000
	1–2 times	58 (10.1%)	225 (39.8%)	28 (6.5%)		
	More than 3 times	14 (2.4%)	118 (20.8%)	2 (0.5%)		
	Books Not at all	382 (66.6%)	163 (28.8%)	385 (89.3%)	469.385	0.000
	1–2 times	156 (27.2%)	192 (33.9%)	38 (8.8%)		
	More than 3 times	36 (6.3%)	211 (37.3%)	8 (1.9%)		
	Internet Not at all	34 (5.9%)	34 (6.0%)	163 (37.8%)	303.226	0.000
	1–2 times	207 (36.1%)	167 (29.5%)	160 (37.1%)		
	More than 3 times	333 (58.0%)	365 (64.5%)	108 (25.1%)		

### Associations between mothers’ individual characteristics, media use, and decisional authority and vaccinating their children

Table 2 shows the results for the social determinants of whether or not East Asian mothers used national immunization programs for their children. In Model I, after we adjusted for the respondents’ socioeconomic characteristics, mothers with high health literacy were more likely to have their children immunized (aOR: 1.210; 95% CI: 1.067–1.373). In Model II, to which we added media use, getting health information from the radio increased the likelihood that the women would immunize their children (aOR: 1.518; 95% CI: 1.013–2.276). In Model III, to which we added maternal capacity, mothers who had high decisional authority in terms of their control of the household finances were more likely to immunize (aOR: 1.264; 95% CI: 1.097–1.458). In contrast, in Model IV, high health literacy and radio use were important predictors of East Asian mothers’ likelihood of having their children immunized.

**Table 2:** Associations between mothers’ individual characteristics, media use, and decisional authority and vaccinating their children (n = 1571).

		Model I		Model II		Model III	
		aOR	95% CI	aOR	95% CI	aOR	95% CI
Constant		3.807		4.639		4.313	
Socioeoconomic	Education	1.142	(0.849–1.535)	1.164	(0.863–1.570)	1.159	0.855–1.571
Status	Income	0.995	(0.935–1.058)	0.984	(0.924–1.048)	0.987	0.926–1.052
Public Health	Self-efficacy	1.084	(0.947–1.240)	1.118	(0.962–1.300)	1.242*	1.043–1.478
Capacity	Health literacy	1.210**	(1.067–1.373)	1.217**	(1.071–1.382)	1.203**	1.054–1.373
Media	Television			1.027	(0.805–1.312)	1.054	0.822–1.352
Use	Radio			1.518*	(1.013–2.276)	1.577*	1.040–2.393
	Newspaper			0.933	(0.662–1.314)	0.926	0.651–1.319
	Books			0.854	(0.652–1.119)	0.887	0.671–1.173
	Internet			0.843	(0.670–1.060)	0.828	0.657–1.043
Decisional Authority						1.264***	1.097–1.458

Adjusted for age, education, and monthly income; bold values indicate statistical significance (p < .05).

aOR: adjusted odds ratio; CI: confidence interval

p < .05*, p < .01**, p < .001***

### Differences in social determinants of mothers’ vaccination of their children by nationality among East Asian countries

Table 3 shows the results for the differences predictors of mothers’ taking advantage of national immunization programs by nationality (South Korea, China, or Japan) (S1 and S2 Tables). In Model III, no social determinant affected whether or not mothers immunized their children in any of the three countries. In South Korea, mothers who watched TV for health information were significantly more likely to have their children immunized (aOR: 1.670; 95% CI: 1.008–2.766). In China, mothers who either listened to the radio (aOR: 1.670; 95% CI: 1.008–2.766) or had high decisional authority (aOR = 1.540; 95% CI: 1.192–1.990) were more likely to have their children immunized. In Japan, mothers who had both high health literacy (aOR: 1.337; 95% CI: 1.096–1.631) and high decisional authority (aOR: 1.391; 95% CI: 1.075–1.800) were more likely to immunize their children. Unusually, in China, mothers who used the Internet for information were less likely to have their children immunized (aOR: 0.546).

**Table 3:** Differences in social determinants of mothers’ vaccination of her their children by nationality among East Asian Asia countries.

		South Korea (n = 574)						China (n = 566)						Japan (n = 431)					
		Model I		Model II		Model III		Model I		Model II		Model III		Model I		Model II		Model III	
		aOR	95% CI	aOR	95% CI	aOR	95% CI	aOR	95% CI	aOR	95% CI	aOR	95% CI	aOR	95% CI	aOR	95% CI	aOR	95% CI
Constant		13.02		18.776		15.324		1.471		1.771		2.838		3.016		.000		.000	
Socioeconomic	Education	.760	(.407–1.418)	.826	(.440–1.551)	.857	(.450–1.631)	1.433	(.729–2.818)	1.734	(.860–3.499)	1.366	(.668–2.795)	1.107	(.710–1.727)	1.070	(.678–1.688)	1.129	(.711–1.791)
status	Income	1.076	(.927–1.250)	1.080	(.929–1.257)	1.097	(.941–1.279)	1.041	(.939–1.154)	.988	(.879–1.111)	.972	(.859–1.099)	.986	(.862–1.127)	1.005	(.878–1.151)	.985	(.857–1.131)
Public health	Self-efficacy	1.094	(.788–1.518)	1.155	(.819–1.629)	1.157	(.790–1.694)	1.050	(.777–1.419)	1.058	(.771–1.450)	1.189	(.829–1.705)	1.014	(.786–1.309)	1.062	(.816–1.383)	.936	(.691–1.268)
capacity	Health literacy	1.084	(.821–1.432)	1.093	(.822–1.453)	1.074	(.802–1.437)	1.331*	(1.053–1.683)	1.290*	(1.012–1.646)	1.168	(.886–1.540)	1.320**	(1.090–1.597)	1.358**	(1.117–1.652)	1.337**	(1.096–1.631)
Media use	Television			1.585	(.962–2.610)	1.670*	(1.008–2.766)			.952	(.681–1.329)	.955	(.673–1.355)			.843	(.539–1.320)	.799	(.505–1.264)
	Radio			.618	(.261–1.465)	.567	(.234–1.375)			1.599	(.994–2.572)	1.765*	(1.070–2.912)			.843	(.539–1.320)	.799	(.505–1.264)
	Newspaper			1.519	(.628–3.675)	1.512	(.605–3.777)			1.313	(.878–1.964)	1.254	(.826–1.903)			.930	(.319–2.712)	.881	(.299–2.591)
	Books			.752	(.435–1.299)	.825	(.467–1.458)			1.181	(.821–1.699)	1.153	(.791–1.681)			1.331	(.604–2.930)	1.314	(.588–2.934)
	Internet			.748	(.513–1.091)	.752	(.515–1.098)			.537	(.384-.750)	.546***	(.387-.773)			.785	(.594–1.038)	.757	(.570–1.006)
Decisional authority						1.315	(.954–1.813)					1.540***	(1.192–1.990)					1.391*	(1.075–1.800)

Adjusted for age, education, and monthly income; bold values indicate statistical significance (p < .05).

aOR: adjusted odds ratio; CI: confidence interval

p < .05*, p < .01**, p < .001***

## Discussion

Although maternal capacity and empowerment can be ambiguous concepts, in fact, their theoretical and operational definitions have been well developed. Of course, decision-making, autonomy, and conjugal power structures are complex issues, so that there exist some differences in the definitions according to the literature [28]. Maternal capacity has been reported to have a positive effect on child health in the national-level data sets of underdeveloped and developing countries [29–33], although research on a concrete definition and how it helps specific health issues has been insufficient. Moreover, the understanding of the social contexts of mothers in diverse countries and cultures has been inadequate as well. Consequently, in the present study, we explored the effects of the decisional authority of mothers in their 20s-40s living in three East Asian countries, South Korea, China, and Japan, on whether or not they had vaccinated their underage children.

The study revealed that for East Asian mothers, decisional authority, self-efficacy, and health literacy all increased the likelihood that they would vaccinate their children, and women were also more likely to vaccinate who searched for health information by listening to the radio. However, there were considerable differences in these characteristics by country. After we adjusted for individual socioeconomic characteristics including age, educational attainment, and household income, decisional capacity was an important predictor of vaccination among Japanese and Chinese women, and using media to seek health information was for vaccination among South Korean and Chinese women. Moreover, the South Korean mothers preferred to receive their health information from television, and Chinese mothers preferred the radio, another difference by country.

The present study has many implications for strategies to increase worldwide vaccination rates under the GVAP. First, the results suggest that behavioral science approaches are necessary for mothers in addition to addressing vaccination constraints such as socioeconomic status or low access). It is very difficult to increase socioeconomic status individually in order to increase vaccination rates. However, areas where socioeconomic status has a strong effect lack sufficient public health systems, which is likely to decrease individuals’ vaccination rates. For example, in Africa, vaccination rates rose when access to medical suppliers and clean drinking water increased [10, 34]. However, it is difficult to make immediately visible reforms to public health systems. We therefore are interested in the issue of maternal capacity, from which considerable effects can be expected even with limited financial resources, and the importance of the issue is empirically revealed by the results of the present study. Similarly, earlier studies that conducted systematic literature reviews using Human Development Index (HDI) scores highlighted possible positive associations between HDI scores and vaccination rates [35].

Second, this study shows that it is necessary to establish locally tailored strategies in order to raise vaccination rates under the GVAP. In advanced countries, correlations between individual socioeconomic factors and vaccination rates are low when access to vaccines is high [19]. However, in addition to public health infrastructures, individual beliefs, perceived risks, and low self-efficacy or capacity can lower children’s vaccination rates. Consequently, it is also necessary to address both domestic and international disparities in vaccine compliance. Even mothers who live in advanced countries but have low socioeconomic status may not be able to vaccinate their children because of low health literacy and low maternal capacity. It is therefore necessary to address the various socioeconomic constraints to vaccination [17, 36, 37].

Third, this study also shows that social contexts must be taken into consideration in order to raise vaccination rates. We analyzed three countries in East Asia, and the social determinants of the mothers’ vaccinating their children differed considerably by nation. The four vaccines that served as the dependent variables were objects of national vaccination programs in all three countries, and cost was not an obstacle. One study found that mothers were less likely to fully vaccinate their children when they were younger or lived in rural areas [22, 38], and thus, we controlled for these variables in our model. Ideally, the reluctance to vaccinate should be reduced through in-depth approaches to sociocultural factors that determine individual attitudes [38, 39].

Several limitations should be noted. We used a cross-sectional data set for this study, but we could not consider all of the possible social determinants of decision-making behavior regarding vaccination. Nevertheless, the findings will help to derive local community-level intervention strategies for increasing the overall vaccination rates in East Asia.

This study revealed that for East Asian mothers, decisional authority, self-efficacy, and health literacy all increased the likelihood that they would vaccinate their children. Furthermore, women who searched for health information through media such as the radio were more likely to vaccinate their children. However, when elaborate analyses were conducted by country, there were considerable differences in those characteristics by country. Therefore, this study showed that it is necessary to establish locally tailored strategies in order to raise vaccination rates in the Global Vaccine Action Plan. This study also showed that social contexts must be taken into consideration in order to raise vaccination rates. Health communication strategies that transparently convey vaccination effects and expected risks to vulnerable maternal groups with low decisional capacity, health literacy, and self-efficacy through various media channels are among such strategies [16, 40–42]. Moreover, it is also necessary to develop standardized indices of mothers’ health capacity such as the HDI and to continuously monitor the effects of intervention strategies and changes in vaccination rates [43].

## Supporting information

S1 File Raw data. (XLS)

S2 File Questionnaire (Chinese). (PDF)

S3 File Questionnaire (Japanese). (PDF)

S4 File Questionnaire (Korean). (PDF)

S1 Table Reg-1. (PDF)

S2 Table Reg-2. (PDF)
