# Supplementary material for: The effect of maternal decisional authority on children's vaccination in East Asia
Source: PLoS One. 2018 Jul 12;13(7):e0200333. doi: 10.1371/journal.pone.0200333 (PMC6042723; doi:10.1371/journal.pone.0200333)
Supplement: S2 File — (PDF) [file pone.0200333.s002.pdf]

[调查问卷]

非药物性仲裁和疫苗适应度的影响涉及的
交流不平等因素分析及新型感染病对应体系的开发
Ver 3.0

您好!

本次调查是在韩国研究财团的支援下进行「非药物性仲裁和疫苗适应度的影响涉及的交流不平等因素」的。

本研究是在探索对接种疫苗所产生的社会背景因素，以母性为中心提高疫苗接种率，强化非药物性仲裁和缓解社会阶层间的沟通不平等为目的，而进行的亚洲与国际的比较研究。以调查问卷的形式收集到的内容，遵守韩国的「统计法」第33条的规定以无记名的方式进行收集信息，所收集的信息除了做学术研究和统计分析以外决不会做其它用处。

请根据真实情况把您的想法和经验如实的进行回答。事先声明一下，您所参加的调查问卷是在您完全自愿的前提下进行的，如果您不愿意可以不参加或是不回答。您所参与的调查问卷资料只用在国家的学术研究调查中，并且会将分析结果刊登在专业的学术杂志上。

您的调查问卷会用在提高韩国和亚洲主要国家疫苗接种率和非药物性仲裁活动中，以及预防和早期对应全世界增加的新型病毒之中。

谢谢!

2017年 1月

同德女子大学 自然科学大学 保健管理学科
丁珉秀 教授 (+82-2-940-4483)

A. 对母性的基初问题

A1. 您的性别是?

① 男性 -----> 설문조사 종료
② 女性

A2. 您的年龄是 ?(   )岁
[20~40세까지만 조사 참여 가능]

A2-1. 您目前的居住地是？

① 上海 -----> 설문조사 종료
② 北京
③ 天津 -----> 설문조사 종료
④ 广州 -----> 설문조사 종료
⑤ 深圳 -----> 설문조사 종료
⑥ 其他 地区 -----> 설문조사 종료

A3. 您的婚姻状况 ?

① 已婚(包括事实婚，同居)
② 分居(以离婚为目的)
③ 死别/死踪 -----> 설문조사 종료
④ 离婚 -----> 설문조사 종료
⑤ 未婚 -----> 설문조사 종료

A4. 您有妊娠经验吗?

① 有
② 没有 -----> B1로 이동

A5. 您有分娩经验吗?

① 有
② 没有 -----> B1로 이동

A5-1. 您生过几男几女?

名

A5-2. 您有未成年(未满１８周岁)子女吗?

① 有
② 没有

B. 利用媒体

B1. 下面的媒体您<u>一天平均</u>用多长时间?

		不用	不到<br>10分	10-30<br>分	30-60<br>分	1-2个<br>小时	2-3个<br>小时	3-5个<br>小时	5个小时<br>以上
1	看电视	①	②	③	④	⑤	⑥	⑦	⑧
2	听收音机	①	②	③	④	⑤	⑥	⑦	⑧
3	读报纸	①	②	③	④	⑤	⑥	⑦	⑧
4	用智能手机上网	①	②	③	④	⑤	⑥	⑦	⑧
5	用电脑上网	①	②	③	④	⑤	⑥	⑦	⑧

B2. 您平时认真去寻找<u>**健康信息**</u>吗?

① 非常认真　② 认真　③ 一般
④ 不感兴趣　⑤ 几乎不找

B3. <u>**过去的一周里**</u>您用下面的媒体寻找过<u>自己还是家人</u>的<u>**健康信息**</u>吗?

		没有	1-2次	3-4次	5次 以上
1	电视	①	②	③	④
2	收音机	①	②	③	④
3	报纸	①	②	③	④
4	书籍	①	②	③	④
5	网络	①	②	③	④

B4. 往往有些人寻找健康信息时会遇到麻烦，您在下面的情况中遇到过多少<u>**麻烦**</u>?

		一点儿<br>不难	难一点	非常难
1	信息太多	①	②	③
2	信息是否正确无法搞清楚	①	②	③
3	无法搞清楚信息是否最新的	①	②	③
4	无法知道信息是否与我有关	①	②	③
5	可用的信息中利用了太多专门用语	①	②	③
6	很难利用网上探索工具和软件	①	②	③
7	难以接近网络	①	②	③

C. 产前管理和医疗利用

C1. 您在上个月里去几次医院门诊治疗过?

① 没去过医院
② 去过1次
③ 去过2次
④ 去过3次
⑤ 去过4次
⑥ 去过5次以上或住院

C2. 过去的一年里您有必要到医院去治疗或检查，可是并没得到过机会，是吗?

① 是(有过一次以上)
② 不是(一次也没有)

[A5에서 1인 사람만 응답, 그 외에는 C6로 이동]

C3. 您最近妊娠期间为了产前管理去过几次医院?

次

C4. 最近您分娩过未熟儿或早产儿吗?

① 有
② 没有

C5. 最近分娩的婴儿体重是多少公斤?

	.		

KG

C6. 您的身高是多少厘米?

CM

C7. 您的体重是多少公斤?

KG

D. 接种疫苗

[A5-2에서 ①예(19세 이하의 자녀가 있는 경우)인 경우에만 응답, 그 외에는 D7로 이동]
[해당 상단 안내문은 D1~D6까지 모두 노출함]

<若有未成年子女(未满１８周岁)的话, 请回答下面问题。多子女家庭请以年幼子女为准回答。>

D1. 为了预防**结核病**给您的子女接种过BCG吗?
- ① 有
- ② 没有

D2. 为了预防**小儿麻痹症**给您的子女接种过POLIO吗?
- ① 有
- ② 没有

D3. 为了预防**白喉，破伤疯，百日咳**，给您的子女接种过DPT吗?
- ① 有
- ② 没有

D4. 为了预防**麻疹(红疫)，痄腮，风疹**给您的子女接种过疫苗吗?
- ① 有
- ② 没有

D5. 为了预防流行性乙型脑炎，给您的子女接种过**流行性乙型脑炎**疫苗吗?
- ① 有
- ② 没有

[D1~D5에서 모두 ② 아니오 선택 시 D7로 이동]

D6.给您的子女接种以上的预防疫苗(结核，**小儿麻痹症，白喉，破伤疯，百日咳**，**麻疹(红疫)，痄腮，风疹**，行性乙型脑炎)时费用是怎么处理的?
- ① 是国家免费接种的。
- ② 本人付一小部分的费用
- ③ 本人付一半的费用
- ④ 本人付大部分的费用
- ⑤ 是本人加入的民间保险公司负责的

D7. 过去一年里您接种过流感疫苗吗?(您本人)?
- ① 有
- ② 没有

E. 健康水平和健康行为

E1. 您的健康水平怎么样?

① 非常不好
② 不好
③ 一般
④ 好
⑤ 很好

[A5에서 1인 사람만 응답, 그 외에는 E3로 이동]
<家里有子女的话, 请回答下面问题。多子女家庭请以年幼子女为准回答。>
E2. 您子女的健康水平怎么样?

① 非常不好
② 不好
③ 一般
④ 好
⑤ 很好

E3. 您现在吸烟吗?

① 吸烟
② 不吸烟 -----> E4로 이동

E3-1. 您的平均一天吸烟量?

① 半合以内　② 一合　③ 一合半　④ 两合以上

E4. 最近一年里您喝过一杯以上的酒吗?

① 有
② 没有 -----> F1로 이동

E4-1. 您经常喝酒吗?

① 一个月不到一次
② 一个月一次
③ 一个月2-4次
④ 一个星期2-3次
⑤ 一个星期4次以上

E4-2. 您一次喝多少酒? (不分白酒，啤酒)

① 1-2杯　② 3-4杯　③ 5-6杯　④ 7-9杯 以上

F. 危险行为及自我效能感

F1. 您是否很想参加下面的活动?

		很不想	不想	想	非常想
1	想攀岩	①	②	③	④
2	即使知道有危险，也想去做充满惊险刺激的事情	①	②	③	④
3	想去做类似滑水和冲浪一样特别过瘾的活动	①	②	③	④
4	想跳降落伞	①	②	③	④
5	想在山顶快速滑雪	①	②	③	④
6	想跳蹦极	①	②	③	④
7	想在游乐园坐过山车之类的惊险的游乐设施	①	②	③	④
8	想跟同龄的飞车族在一起玩耍	①	②	③	④

F2. 请把<u>**您的想法**</u>给表示一下。

		完全<br>不对	不对	一般	对	非常对
1	我有信心	①	②	③	④	⑤
2	我容易放弃	①	②	③	④	⑤
3	我会按计划把工作做好	①	②	③	④	⑤
4	我容易躲避困难	①	②	③	④	⑤
5	当初知道有错，也要做到底	①	②	③	④	⑤
6	我只要有目标就能去实现	①	②	③	④	⑤
7	我觉得有一点麻烦就不动手	①	②	③	④	⑤
8	我只要有事要做就立即行动	①	②	③	④	⑤
9	发生意外就不知所措	①	②	③	④	⑤

G. 母性力量

G1. 您怎样处理下面问题？请您如实的回答。

		本人	和对方一起	对方	其他人
1	对您收入的用途有谁来决定?	①	②	③	④
2	你们家里谁的收入高?	①	②	③	④
3	在您家里谁来决定对方收入的用途?	①	②	③	④
4	谁有权决定您对医疗的利用?	①	②	③	④
5	在您家里买家用品时有谁来做主?	①	②	③	④
6	在您家里探亲或去看望家人时有谁来做主?	①	②	③	④

G2. 请您如实的回答，您对生活的主导力量是什么?

		决不会	不会	一般	对	非常对
1	我能自由的选择自己的人生	①	②	③	④	⑤
2	我拥有女性的优点	①	②	③	④	⑤
3	我做为女人对自己的外貌满有信心	①	②	③	④	⑤
4	我为自己的健康确保时间	①	②	③	④	⑤
5	坚持适合我的运动	①	②	③	④	⑤
6	从配偶听说过辱骂和暴言	①	②	③	④	⑤
7	与配偶吵架中被挨打过	①	②	③	④	⑤
8	在配偶的强迫下发生过性关系	①	②	③	④	⑤

H. 健康文解力

H1. 请您如实的回答对健康信息是否理解并利用和沟通。

		能	难	很难
1	在医院里提供的表格(例:手术同意书，诊疗说明书，服药说明书)能理解或填写吗?	①	②	③
2	您能看懂或填写患者的一览表吗?	①	②	③
3	医生提供的医疗信息印刷物您能理解吗?	①	②	③
4	预约诊疗的方法您知道吗?	①	②	③
5	能向医生提问您所不理解的健康信息吗?	①	②	③

H2. 请您如实的回答关于**健康信息**的以下问题。

		绝对不可能	不可能	一般	可能	非常可能
1	我知道在网上怎样找有用的健康信息	①	②	③	④	⑤
2	我知道在网上找到的健康信息的用法	①	②	③	④	⑤
3	我能分别清楚在网上找到的健康信息是否有用	①	②	③	④	⑤
4	我为了决定有关健康的问题，能利用网络信息	①	②	③	④	⑤

H3. 平时您对医疗统计数据的理解有多难?

① 非常容易　② 容易　③ 难　④ 很难

H4. 在下面得疾病的危险性最大的数字是哪一个?

① 100 分之 1　② 1,000分之1　③ 10分之1

H5. 人们常常用两种语言来表达某种事情的发生率，例如"偶尔发生"，和"5%的可能性"这两种表方式中您喜欢哪一种?

① 喜欢词汇(偶尔发生)的表达方式

② 喜欢数字(5%的可能性)的表达方式

③ 两种都可以

I. 知觉危险

[A5에서 1인 사람만 응답, 그 외에는 I3로 이동]

I1. 下面是问您对知觉危险的问题，有可能发生在<u>您的子女</u>身上的地方请标(√)号。

		很低	比<br>平均低	平均	比<br>平均高	非常高
1	今年冬天得感冒的可能性有多大?	①	②	③	④	⑤
2	今后一年内，因病要住院的可能性有多大?	①	②	③	④	⑤
3	今后一年内，骨折的可能性有多大?	①	②	③	④	⑤
4	今后一年内，得水痘的可能性有多大?	①	②	③	④	⑤
5	今后一年内，发高烧的可能性有多大?	①	②	③	④	⑤

I2. 下面的情况发生在<u>他人子女</u>的可能性中请标(√)号。

		很低	比<br>平均低	平均	比<br>平均高	非常高
1	今年冬天得感冒的可能性有多大?	①	②	③	④	⑤
2	今后一年内，因病要住院的可能性有多大	①	②	③	④	⑤
3	今后一年内，骨折的可能性有多大?	①	②	③	④	⑤
4	今后一年内，得水痘的可能性有多大?	①	②	③	④	⑤
5	今后一年内，发高烧的可能性有多大?	①	②	③	④	⑤

I3. 在下面找一下适合您的项目中请标(√)号。

		绝对<br>没有	很低	与一般人<br>差不多	比较高	非常高
1	您觉得在一年之内得流行性传染病的可能性有多大?	①	②	③	④	⑤
2	您觉得在一年之内得癌的可能性有多大?	①	②	③	④	⑤
3	您觉得一年之内要离婚的可能性有多大?	①	②	③	④	⑤

J. 非药物性仲裁

[단, 해당 그림은 중국어, 일본어의 경우 하단의 텍스트는 없이 진행]

J1. 下面是**WHO提示的正确洗手方法**，您平时遵守这个规则吗?

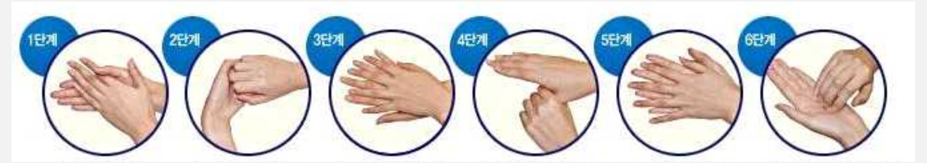

① 几乎不守这个规则
② 一般不守这个规则
③ 一般
④ 守这个规则
⑤ 遵守这个规则

J2. 在平时生活中您遵守下面的预防疾病规则吗?

		决不实行	不怎么实行	一般	最好实行	必须
1	洗手或使用洗手剂	①	②	③	④	⑤
2	躲避与传染病患者的接触	①	②	③	④	⑤
3	不要用手摸眼睛，鼻子，嘴等等	①	②	③	④	⑤
4	传染病流行时不出家门	①	②	③	④	⑤
5	少去人多的场所	①	②	③	④	⑤
6	要戴口罩	①	②	③	④	⑤

J3. 您相信下面的各个信息源吗?

		决不相信	不相信	相信	非常相信
1	电视	①	②	③	④
2	收音机	①	②	③	④
3	报纸	①	②	③	④
4	网络	①	②	③	④
5	政府(保健福祉部，疾病管理本部)	①	②	③	④
6	医生或保健专家	①	②	③	④
7	得过该疾病的家人或朋友	①	②	③	④

K. 有关健康知识

K1. 下面是有关<u>艾滋病</u>的问题。请您说出对错。

		对	错
1	与艾滋病患者共用一个水杯会感染艾滋病的	①	②
2	与艾滋病患者共餐会感染艾滋病的	①	②
3	蚊子也可以感染艾滋病的	①	②
4	性交时用避孕套可以预防艾滋病的感染	①	②
5	适当的治疗和 健康管理使艾滋病患者能活到30年以上	①	②

K2. 如果我国<u>成年人对健康知识的水平平均为50分</u>的话，<u>您的分数</u>是多少?

0分	10分	20分	30分	40分	国民平均50分	60分	70分	80分	90分	100分
1	2	3	4	5	6	7	8	9	10	11

K3. 下面是对<u>新型感染病</u>的调查，适合您的项目中请做标(√)号。

		绝对不是	不是	一般	是	绝对是
1	您可以正确地说明正流行的新型感染病吗?	①	②	③	④	⑤
2	您最近对正流行的感染病，通过新闻等报道观注过进行过程吗?	①	②	③	④	⑤
3	您觉得今后我国发生新型感染病的可能性大吗?	①	②	③	④	⑤
4	您认为WHO世界保健机构对最近流行的感染病采取的措施妥当吗?	①	②	③	④	⑤
5	您认为政府对最近流行的感染病采取的措施妥当吗?	①	②	③	④	⑤

K4. 下面是对<u>新种流行性感冒</u>看法。请把您的看法指出来。

		对	不对
1	新种流行性感冒的病原体是病毒不是细菌	①	②
2	新种流行性感冒可以通过接种预防	①	②
3	得新种流行性感冒会有发烧(37.8℃)，流鼻涕，咽喉痛，咳嗽等症状	①	②
4	新种流行性感冒主要以患者的唾液和飞沫传染的	①	②

5	患有新中流行性感冒而不治疗大部分患者都会死亡	①	②
6	勤洗手对预防新种流行性感冒有很大的效果	①	②

K5. 请把您对**中东呼吸综合征(MERS)的想法指出来。**

		对	错
1	MERS是没有症状的潜伏期中也可以传染	①	②
2	MERS 在健康的人与人之间是不容易传染的	①	②
3	MERS主要有发烧，咳嗽，呼吸困难等症状	①	②
4	去接待过MERS患者的医院也有可能被传染	①	②
5	接触过MERS患者，必须从接触日起在家或指定的医疗机关里隔离7天	①	②
6	少去人多拥挤的场所能助于预防MERS的传染	①	②
7	回国后14天之内，如果有发烧，呼吸困难等症状立即向保健所等单位申报	①	②

K6. 请把您对**塞卡病毒(Zika virus)的想法指出来。**

		对	错
1	白线林蚊子能传染塞卡病毒	①	②
2	塞卡病毒不会因输血而增加传染	①	②
3	如果怀疑患，塞卡病毒的育龄妇女从塞卡病毒发生国回国之后， 至少延期两个月以后妊娠为好。	①	②
4	被塞卡病毒感染会导致死亡	①	②
5	塞卡病毒主要通过唾液等飞沫传染	①	②
6	被塞卡病毒感染的话，最长在两周之内会有疹，发烧，关节痛，肌肉痛，眼睛充血等症状	①	②
7	孕妇(特别是妊娠初期)被塞卡病毒感染的话，会引起胎儿小头症	①	②

L. 健康生活的质量和社会的支持

L1. 下面是对您健康状态的问题，适合您的项目中请标(√)号。

		非常正确	正确	不正确
1	我可以自己走路	①	②	③
2	我可以自己洗澡或穿衣服	①	②	③
3	我可以正常活动	①	②	③
4	我没有疼症和不舒服之感。	①	②	③
5	我没有不安和忧郁	①	②	③

L2. 在下面适合您的项目中请标(√)号。

		总有	几乎总有	有	偶尔有	没有
1	过去一个月里您在精神上，身体上有过难以承受感觉吗?	①	②	③	④	⑤
2	过去一个月里您在您的生活原则中觉得有过什么挫折吗?	①	②	③	④	⑤
3	过去一个月里您有过作为一个人最基本的欲望都没满足的感觉吗?	①	②	③	④	⑤
4	过去一个月里您因不明确的未来而感到过不安吗?	①	②	③	④	⑤
5	过去一个月里因要做的事情太多，而错过更重要的事情吗?	①	②	③	④	⑤

L3. 您对下面的文章同意吗?

		决不	不	同意	赞成
1	周围的人积极帮助邻居	①	②	③	④
2	邻居可以相信	①	②	③	④
3	邻居们常见面或常聚会	①	②	③	④

L4. 您除了家人以外和朋友们常见面吗?

① 一个月不到1次　② 一个月1次　③ 一个月2-3次　④ 1周 1次
⑤ 1周 2-3次　⑥ 几乎每天

L5. 一周内您常独自一人吃饭吗?

① 几乎每天　② 一周5次以上　③ 一周3-4次　④ 一周1-2次
⑤ 几乎没有

M. 应答者的一般特性

M1. 您的最高学历是?

① 小学未毕业
② 中学毕业
③ 高中毕业
④ 大学毕业
⑤ 在读大学院以上

M2. 您现在做有所得的经济活动吗?

① 有
② 没有 -----> M3로 이동

M2-1. 您的职位是什么?

① 长期工
② 临时工

M3. <u>您的配偶</u>的最高学历是?

① 小学未毕业
② 中学毕业
③ 高中毕业
④ 大学毕业
⑤ 在读大学院 以上

M4. <u>您的配偶</u>现在做有所得的经济活动吗?

① 有
② 没有

M5. (工资，月薪，社会保障或退休年金，亲属的帮组等所有的收入都包括在内时)
2016年 <u>您家的总收入</u>是多少? 请说税前收入。

① 不到 60,000元
② 60,000元 以上 不到 120,000元
③ 120,000元 以上 不到 180,000元
④ 180,000元 以上 不到 240,000元
⑤ 240,000元 以上 不到 300,000元
⑥ 300,000元 以上 不到 360,000元
⑦ 360,000元 以上 不到 420,000元
⑧ 420,000元 以上 不到 480,000元
⑨ 480,000元 以上 不到 540,000元
⑩ 540,000元 以上 不到 600,000元
⑪ 600,000元 以上

- 谢谢! -
