# Supplementary material for: The effect of maternal decisional authority on children's vaccination in East Asia
Source: PLoS One. 2018 Jul 12;13(7):e0200333. doi: 10.1371/journal.pone.0200333 (PMC6042723; doi:10.1371/journal.pone.0200333)
Supplement: S3 File — (PDF) [file pone.0200333.s003.pdf]

非薬物的仲裁とワクチン順応度に影響をおよぼす
ユミュニケーション不平等要因分析・新種感染病対応体系の開発
Ver 3.0

はじめまして。

当調査は、韓国研究財団の支援を受けて行われる『非薬物的仲裁とワクチン順応度に影響をおよぼすユミュニケーション不平等要因』です。

この研究はワクチン接種に影響をおよぼす社会脈絡的要因を探求し、母性を中心にワクチン接種率を上げ、非薬物的仲裁を強化して社会階層間健康ユミュニケーションの不平等を緩和するため実施されるアジア圏国際比較研究です。アンケートを通して集められた応答内容は韓国の『統計法』に基づき完全無記名として処理され、学術的研究と統計分析以外の目的には使用されません。

事実に基づいたあなたの意見と経験を率直にお聞かせください。アンケートは自律的に行われますので、お望みでない場合ご返答くださらなくてもいいです。ご返答くださったアンケート資料は国家の学術的研究調査のみに活用され、分析結果は専門の学術誌に掲載されます。

あなたの応答内容はアジア圏主要国家のワクチン接種率を向上し、非薬物的仲裁活動を強化することにより、前世界的に増加している新種感染病の予防・早期対応にとても貴重に使われます。ありがとうございます。

2017年 1月

同徳女子大学校 自然科学大学 保健管理学科
ジョン・ミンス教授 (+82-2-940-4483)

A. 母性に対する基礎質問

A1. あなたの性別

① 男性 ------> 설문조사 종료

② 女性

A2. あなたの年齢 (　　　　) 歳

[20~40세까지만 조사 참여 가능]

A2-1. どちらの地域にお住まいですか。

① 東京

② 東京外 ------> 설문조사 종료

A3. あなたはご結婚なさっていますか。

① 既婚(同棲中の事実婚も含める)

② 既婚だが、離婚前提の別居中

③ 死別/失踪 ------> 설문조사 종료

④ 離婚 ------> 설문조사 종료

⑤ 未婚 ------> 설문조사 종료

A4. 妊娠の経験はありますか。

① はい

② いいえ ------> B1로 이동

A5. 出産の経験はありますか。

① はい

② いいえ ------> B1로 이동

A5-1. あなたは何人の子供を産みましたか。

名

A5-2. 未成年(**満19歳未満**) の子供がいますか。

① はい

② いいえ

B. メディア活用

B1. あなたは**1日平均的**に以下のメディアをそれぞれどのぐらい使用していますか。

		全く使用しない	10分未満	10分以上 - 30分未満	30分以上 - 60分未満	1時間以上 - 2時間未満	2時間以上 - 3時間未満	3時間以上 - 5時間未満	5時間以上
1	TVの視聴	①	②	③	④	⑤	⑥	⑦	⑧
2	ラジオ聴取	①	②	③	④	⑤	⑥	⑦	⑧
3	新聞を読む	①	②	③	④	⑤	⑥	⑦	⑧
4	スマホでウェブ検索	①	②	③	④	⑤	⑥	⑦	⑧
5	パソコンでウェブ検索	①	②	③	④	⑤	⑥	⑦	⑧

B2. あなたは普段どれぐらい積極的に**健康情報**を探しますか。

① 熱心に探す　② 探すほうだ　③ まあまあだ
④ 探さないほうだ　⑤ あまり探さない

B3. あなたはこの**一週間**以下のメディアを通して、**本人または家族の健康情報**を探したことがありますか。

		探していない	1-2回	3-4回	5回以上
1	TV	①	②	③	④
2	ラジオ	①	②	③	④
3	新聞	①	②	③	④
4	本	①	②	③	④
5	ネット	①	②	③	④

B4. 人は健康情報を探すとき時々困難を覚えます。あなたは以下の各状況でどれだけ困難を覚えましたか。

		全然なかった	あった	とてもあった
1	情報が多すぎる。	①	②	③
2	情報が確かなのか分からない。	①	②	③
3	情報が最新のものか分からない。	①	②	③
4	情報が自分に合っているものか分からない。	①	②	③
5	探した情報に専門的な用語が含まれ過ぎていた。	①	②	③
6	オンライン探索の道具やソフトウェアが使いづらい。	①	②	③
7	ネットを自由に使いづらい。	①	②	③

C. 産前管理と医療の利用

C1. あなたはこの<u>一ヶ月間</u>病院や医院から<u>外来診療</u>を受けたことがありますか。あるなら何回ですか。

① この一ヶ月間外来診療を受けたことがない
② 1回訪問
③ 2回訪問
④ 3回訪問
⑤ 4回訪問
⑥ 5回以上訪問または入院

C2. あなたはこの1年間病院・医院で治療や検査を受ける必要はあったけど、受けられなかったことが一度でもありましたか。

① はい (受けなかったことが一度でもあった。)
② いいえ (受けなかったことが一度もなかった。)

[A5에서 1인 사람만 응답, 그 외에는 C6로 이동]

C3. あなたは<u>一番最近、</u>妊娠期間中産前管理のため何回医療サービスを受けましたか。

回

C4. <u>一番最近、</u>子供が早産、または未熟児で産まれましたか。

① はい
② いいえ

C5. <u>一番最近、</u>ご出産の際、子供の体重は何ｋｇでしたか。
(※ 記入欄はブランクにしないでください)

	.		

KG

C6. あなたの背は何cmですか。

CM

C7. あなたの体重は何kgですか。(※ 記入欄はブランクにしないでください)

KG

D. ワクチン接種

[A5-2에서 ①예(19세 이하의 자녀가 있는 경우)인 경우에만 응답, 그 외에는 D7로 이동]
[해당 상단 안내문은 D1~D6까지 모두 노출함]

＜以下は未成年の子供(満２０歳未満）に関する質問です。未成年の子供が二人以上の場合は、一番最後に生んだ子供を基準にご返答ください。＞

D1. 結核を予防するため、あなたの子供に**BCG予防接種**をしましたか。

① はい
② いいえ

D2. 小児麻痺を予防するため、あなたの子供に**ポリオ(Polio)予防接種**をしましたか。

① はい
② いいえ

D3. ジフテリア、破傷風、百日咳を予防するため、あなたの子供に**DPT予防接種**をしましたか。

① はい
② いいえ

D4. 麻疹、おたふくかぜ、風疹を予防するため、あなたの子供に**麻疹・おたふくかぜ・風疹の予防接種**をしましたか。

① はい
② いいえ

D5. 日本脳炎を予防するため、あなたの子供に**日本脳炎の予防接種**をしましたか。

① はい
② いいえ

[D1~D5에서 모두 ② 아니오 선택 시 D7로 이동]

D6.上記の予防接種（結核、小児麻痺、ジフテリア、破傷風、百日咳、麻疹、おたふくかぜ、風疹、日本脳炎）を行うとき、接種費用の負担はどうされましたか。

① 国で行われる無料接種だった
② 少しだけ自分で払った
③ 半分ぐらい自分で払った
④ ほとんど自分で払った
⑤ 個人的に持っている民間保健から払った

D7. あなたはこの一年間インフルエンザのワクチン接種を受けたことがありますか。

① はい　　② いいえ

E. 健康水準および健康行動

E1. あなたの健康水準はどうですか。

① とても悪い
② 悪い
③ 普通だ
④ 良好
⑤ とても良好

[A5에서 1인 사람만 응답, 그 외에는 E3로 이동]
＜以下はあなたの子供に関する質問です。子供が二人以上の場合は、一番最後に生んだ子供を基準にご返答ください。＞

E2. あなたの子供の健康水準はどうですか。

① とても悪い
② 悪い
③ 普通だ
④ 良好
⑤ とても良好

E3. あなたは現在**タバコ**を吸っていますか。

① はい
② いいえ -----> E4로 이동

E3-1. あなたの**１日の平均的な喫煙量**はどのぐらいですか。

① 半箱以下　② １箱ぐらい　③ １．５箱ぐらい　④ ２箱以上

E4. あなたは**この一年間、1杯以上のお酒**を飲んだことがありますか。

① はい
② いいえ -----> F1로 이동

E4-1. あなたはどのぐらいの頻度でお酒の飲みますか。

① 月に1回未満
② 月に1回ぐらい
③ 月に2-4回ぐらい
④ 週に2-3回ぐらい
⑤ 週に4回以上

E4-2. あなたはどのぐらいお酒を飲みますか。(焼酎，ビール関係なく)

① 1-2杯　② 3-4杯　③ 5-6杯　④ 7-9杯以上

F. 危険行動および自己効能感

F1. あなたは以下の行動をどれぐらいやってみたいとおもいますか。

		全然やりたくない	やりたくない	やってみたい	とてもやってみたい
1	ロッククライミングをやってみたい。	①	②	③	④
2	怖くてもスリル感あふれることをやってみたい。	①	②	③	④
3	水上スキーやサーフィンのようなぞくぞくするスポーツをやってみたい。	①	②	③	④
4	飛行機からパラシュートを持って飛び降りてみたい。	①	②	③	④
5	山の急傾斜からスキーをはいて滑ってみたい。	①	②	③	④
6	バンジージャンプをやってみたい。	①	②	③	④
7	遊園地でジェットユースターに乗ってみたい。	①	②	③	④
8	同い年の暴走族と付き合ってみたい。	①	②	③	④

F2. 以下の各項目についての**<u>あなたの意見</u>**を選んで答えてください。

		全然そうではない	そうではない	普通だ	そうだ	とてもそうだ
1	私には自信がある。	①	②	③	④	⑤
2	私は簡単にあきらめる。	①	②	③	④	⑤
3	私は任された仕事を計画通り全うできる。	①	②	③	④	⑤
4	私は難しいことに挑戦することを避けるほうだ。	①	②	③	④	⑤
5	最初から間違っていることでも最後までやってみる。	①	②	③	④	⑤
6	私は目標を立てれば成就することができる。	①	②	③	④	⑤
7	私は複雑そうなことはやらない。	①	②	③	④	⑤
8	私はやるべきことがあったらすぐそれをはじめる。	①	②	③	④	⑤
9	予想外のことへの対処は難しい。	①	②	③	④	⑤

G. 母性力量

G1. 次は家族の中であなたの意思決定に関する項目です。率直にお答えください。

		あなた	あなたと配偶者がいっしょに	配偶者	他の誰か
1	あなたの収入をどう使うかは主にだれが決めますか。	①	②	③	④
2	あなたとあなたの配偶者の中で収入が多いのはどちらですか。	①	②	③	④
3	配偶者の収入をどう使うかは主にだれが決めますか。	①	②	③	④
4	あなたが医療サービスを利用することについて決定をするのはだれですか。	①	②	③	④
5	家庭内の必要品を買うのは主にだれが決定しますか。	①	②	③	④
6	家族または親戚の家に訪問することは主にだれが決定しますか。	①	②	③	④

G2. 以下は人生についてあなたが持っている主導的力量について聞く項目です。率直にお答えください。

		全然そうではない	そうではない	普通だ	そうだ	とてもそうだ
1	自分の人生で重要な選択を自由にすることができる。	①	②	③	④	⑤
2	私は女性として長所を持っている。	①	②	③	④	⑤
3	私は女性として自分の外見を堂々と表現する。	①	②	③	④	⑤
4	自分の健康管理のための時間を確保しようと努力する。	①	②	③	④	⑤
5	自分に合ってる運動を定期的にやる。	①	②	③	④	⑤
6	配偶者に暴言を言われたことがある。	①	②	③	④	⑤
7	配偶者と口喧嘩の最中、殴られたことがある。	①	②	③	④	⑤
8	断ったのにも関わらず配偶者に性行為を無理に強いられたことがある。	①	②	③	④	⑤

H. 健康文解力

H1. 次はあなたが健康に関する情報を理解・活用しコミュニケーションできるかについて聞く項目です。率直にご返答ください。

		問題ない	少々難しい	結構難しい
1	病院で提供する書式(例: 手術同意書, 診療案内書, 服薬説明書 など)を理解した上で書けますか。	①	②	③
2	患者管理のためのチャートの内容を理解して必要な内容を記入できますか。	①	②	③
3	医者が提供する医療情報が載っているプリントを理解できますか。	①	②	③
4	診療の予約のやり方が分かりますか。	①	②	③
5	知らない健康情報を理解するため医者に質問できますか。	①	②	③

H2. 以下はあなたの<u>**ネットを使用した健康情報の活用**</u>に関する質問です。率直にご返答ください。

		とてもそうではない	そうではない	普通だ	そうだ	とてもそうだ
1	私はネットで有用な健康情報をどこで探せるのかを知っている。	①	②	③	④	⑤
2	私はネットで探した健康情報をどう利用するのかを分かっている。	①	②	③	④	⑤
3	私はネットで探した健康情報が良質のものかどうかの区別ができる。	①	②	③	④	⑤
4	私は健康に関する意思決定のためネットの健康情報を活用することに自信がある。	①	②	③	④	⑤

H3. あなたは普段、保健・医療関連の統計を理解する際に難しく感じますか。

① とても簡単だ　② 簡単だ　③ 難しい　④ とても難しい

H4. 次のうち疾病にかかる危険がもっとも大きいのはどれですか。

① 100/1　② 1,000/1　③ 10/1

H5. 人は二つの言葉を使用して事件が発生する確率について話します。例えば、「たまに発生する。」と「５%の可能性がある。」の中であなたはどちらの表現を好みますか。

① 単語 (たまに発生する。) のほうがいい

② 数字 (５%の可能性がある。) のほうがいい

③ どちらでも構わない

I. 危険知覚

[A5에서 1인 사람만 응답, 그 외에는 I3로 이동]

I1.　下記はあなたの危険知覚に対する質問です。質問の状況があなたの子供に発生する可能性はどれぐらいあると思いますか。マーク(✓)してください。

		とても低い	平均より低い	平均値	平均より高い	とても高い
1	この冬風邪を引く可能性はどれぐらいあると思いますか。	①	②	③	④	⑤
2	今後一年以内に病気で入院する可能性はどれぐらいあると思いますか。	①	②	③	④	⑤
3	今後一年以内に骨折する可能性はどれぐらいあると思いますか。	①	②	③	④	⑤
4	今後一年以内に水痘にかかる可能性はどれぐらいあると思いますか。	①	②	③	④	⑤
5	今後一年以内に高熱になる可能性はどれぐらいあると思いますか。	①	②	③	④	⑤

I2.以下の状況があなたの子供以外の子供たちに発生する可能性はどれぐらいだと思いますか。マーク(✓)してください。

		とても低い	平均より低い	平均値	平均より高い	とても高い
1	この冬風邪を引く可能性はどれぐらいあると思いますか。	①	②	③	④	⑤
2	今後一年以内に病気で入院する可能性はどれぐらいあると思いますか。	①	②	③	④	⑤
3	今後一年以内に骨折する可能性はどれぐらいあると思いますか。	①	②	③	④	⑤
4	今後一年以内に水痘にかかる可能性はどれぐらいあると思いますか。	①	②	③	④	⑤
5	今後一年以内に高熱になる可能性はどれぐらいあると思いますか。	①	②	③	④	⑤

I3. 以下の項目を読んで該当するところにマーク(✓)してください。

		全然ない	とても低い	人並みだ	ちょっと高い	とても高い
1	あなたが1年以内に流行性伝染病にかかる可能性はどれぐらいあると思いますか。	①	②	③	④	⑤
2	あなたが1年以内にガンになる可能性はどれぐらいあると思いますか。	①	②	③	④	⑤
3	あなたが1年以内に現在のご主人と離婚する可能性はどれぐらいあると思いますか。	①	②	③	④	⑤

J. 非薬物的仲裁

[단, 해당 그림은 중국어, 일본어의 경우 하단의 텍스트는 없이 진행]

J1. 以下の絵は**WHOから提案した正しい手の洗い方**です。あなたは普段このような規則を守りますか。

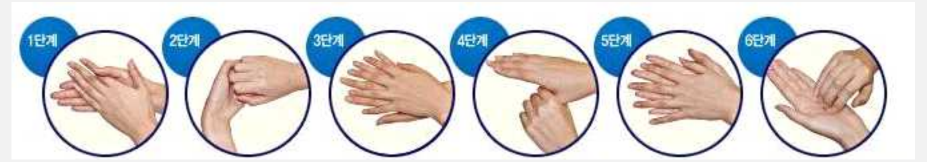

① かなり守らない
② 守らない方だ
③ 普通だ
④ 守る方だ
⑤ よく守る方だ

J2. あなたは普段の生活で以下のような病気予防を実践しますか。

		全然実践しない	あまり実践しない	ほどほどだ	できるだけ実践する	必ず実践する
1	手洗いまたは手の消毒剤の使用。	①	②	③	④	⑤
2	伝染病に感染された人との接触を控える。	①	②	③	④	⑤
3	目・鼻・口などを手で触らない。	①	②	③	④	⑤
4	伝染病が流行っているときの外出を控える。	①	②	③	④	⑤
5	人との接触が頻繁な場所を避ける。	①	②	③	④	⑤
6	マスクの着用。	①	②	③	④	⑤

J3. あなたは下記の情報員をどれぐらい**信頼**していますか。

		全く信頼しない	信頼しない	ちょっと信頼している	とても信頼している
1	TV	①	②	③	④
2	ラジオ	①	②	③	④
3	新聞	①	②	③	④
4	ネット	①	②	③	④
5	政府(保健福祉部、疾病管理本部)	①	②	③	④
6	医者・保健専門家	①	②	③	④
7	当疾病にかかった経験のある家族や知り合い	①	②	③	④

K. 健康関連知識

K1. 次は**エイズ**に関する質問です。各項目についてあなたの意見とあっているものにマーク(✓)してください。

		はい	いいえ
1	エイズ感染者と同じコップを使うとエイズに感染される。	①	②
2	エイズ感染者といっしょに食事をするとエイズに感染される。	①	②
3	エイズは蚊に刺されることで感染されることがある。	①	②
4	エイズは性行為を行うときコンドームを使えば予防できる。	①	②
5	エイズは適切な治療と健康管理があれば３０年以上生きることも可能だ。	①	②

K2. 日本**成人の健康に対する知識水準が平均５０点**だとする場合、**あなたの健康知識の点数**は何点だと思いますか。該当するところにマーク(✓)してください。(10点単位)

0点	10点	20点	30点	40点	国民平均 50点	60点	70点	80点	90点	100点
1	2	3	4	5	6	7	8	9	10	11

K3.
次は**新種感染病**に対する認識度に関する質問です。該当するところにマーク(✓)してください。

		全然違う	違う	普通だ	そうだ	とてもそうだ
1	あなたは新種感染病の流行りについて明確に説明できますか。	①	②	③	④	⑤
2	あなたは最近感染病が流行っているときニュースなどを通じて進行過程に関心を持ちましたか。	①	②	③	④	⑤
3	あなたはこれからも日本に新種の感染病が流行る可能性が高いと思いますか。	①	②	③	④	⑤
4	あなたは最近感染病の流行りに対するWHO(世界保健機構)の対応が適切だったと思いますか。	①	②	③	④	⑤
5	あなたは最近感染病の流行りに対する政府の対応が適切だったと思いますか。	①	②	③	④	⑤

K4.　以下の新種インフルエンザに関する項目についてのあなたの意見と合っているものにマーク(✓)してください。

		そうだ	そうではない
1	新種インフルエンザの病原体はバイ菌ではなくウイルスだ。	①	②
2	新種インフルエンザは予防接種を通して予防できる。	①	②
3	新種インフルエンザにかかると発熱(37.8℃)，鼻水，咽喉痛，咳などの症状が現れる。	①	②
4	新種インフルエンザは主に患者の唾みたいな飛沫によって伝染される。	①	②
5	新種インフルエンザは治療しないとたいてい死亡する。	①	②
6	手をよく洗うと新種インフルエンザの予防になる。	①	②

K5.

以下の**マーズ (中東呼吸器症候群、MERS)**に関するあなたの意見と合っているところにマーク(✓)してください。

		そうだ	そうではない
1	マーズは症状のない潜伏期中にも伝染される。	①	②
2	マーズは防具なしに患者を看護するなどの行為をしない限り簡単に伝染されたりしない。	①	②
3	マーズの主要症状には発熱、咳、呼吸困難などがある。	①	②
4	患者がすでに帰った病院に訪問してもマーズに感染されることができる。	①	②
5	症状が発生した患者と密接に接触した人は最終日から7日間自宅か指定された医療期間に隔離される。	①	②
6	人が多い場所を控えるのはマーズ予防のためのひとつだ。	①	②
7	帰国後１４日以内に発熱、呼吸困難などの異常症状がある場合、すぐ保健所などに連絡しないといけない。	①	②

K6.
以下の<u>ジカウイルス(Zikavirus)</u>についてのあなたの意見と合っているところにマーク(✓)してください。

		そうだ	そうではない
1	ジカウイルスはヒトスジシマカによって伝染される。	①	②
2	ジカウイルスは献血にすることでは伝染されない。	①	②
3	ジカウイルスの予備群の場合、妊娠可能な女性はウイルス発生国から帰国後少なくとも2ヶ月は妊娠を延期しなければならない。	①	②
4	ジカウイルスは感染されたら死に至る可能性が高い。	①	②
5	ジカウイルスは主に唾のような飛沫によって伝染される。	①	②
6	ジカウイルスに感染された場合、最大2週以内に発疹、発熱、関節痛、筋肉痛、目の充血などの症状が現れる。	①	②
7	妊婦が（特に妊娠初期に）感染される場合、胎児小頭症が起る可能性がある。	①	②

L. 健康関連生活の質と社会的支持

L1. 次はあなたの健康状態に対する質問です。該当するところにマーク(✓)してください。

		結構ある	少々ある	ない
1	私は歩くとき支障がない	①	②	③
2	私はお風呂に入ったり服を着るとき支障がない	①	②	③
3	私は日常生活をすることに支障がない	①	②	③
4	私は痛みや不便がない	①	②	③
5	私は不安だったり憂鬱だったりしない	①	②	③

L2. 以下の質問を読んでマーク(✓)してください。

		いつもあった	よくあった	結構あった	たまにあった	全然なかった
1	この一ヶ月、生きていくのに精神的・身体的に耐えづらいと思ったことがありますか。	①	②	③	④	⑤
2	この一ヶ月、自分の生活信念に従って生きようとして挫折を覚えたことがありますか。	①	②	③	④	⑤
3	この一ヶ月、人間として基本的な欲求が満たされてないと感じたことがありますか。	①	②	③	④	⑤

4	この一ヶ月、未来が不確かに思えたり、不安になったことがありますか。	①	②	③	④	⑤
5	この一ヶ月、やることが多すぎて大事なことを忘れたことがありますか。	①	②	③	④	⑤

L3. あなたは以下の文章にどれぐらい同意しますか。

		全然そうではない	そうではない	そうだ	とてもそうだ
1	周りの人たちは隣人を助けることに積極的だ。	①	②	③	④
2	隣人は信用できる。	①	②	③	④
3	隣人とよく会って集まる。	①	②	③	④

L4. あなたは家族以外の友だちとどのぐらいの頻度であっていますか。

① 月に1回未満　② 月に1回　③ 月に2-3回　④ 週に１回
⑤ 週に2-3回　⑥ ほぼ毎日

L5. あなたは週にどれぐらい一人で食事をしますか。

① ほぼ毎日　② 週に5回以上　③ 週に3-4回　④ 週に1-2回
⑤ ほとんど一人で食べない

M. 応答者の一般的特性

M1. あなたの最終学歴は次のうちどれですか。

① 小学校卒業以下
② 中学校卒業
③ 高校卒業
④ 大学卒業
⑤ 大学院在学以上

M2. あなたは現在、所得の発生する経済活動に従事していますか。

① はい
② いいえ ------> M3로 이동

M2-1. あなたが従事する仕事はどんな仕事ですか。

① フルタイムワーカー
② パートタイム・アルバイト

M3. あなたの配偶者の最終学歴は次のうちどれですか。

① 小学校卒業以下
② 中学校卒業
③ 高校卒業
④ 大学卒業
⑤ 大学院在学以上

M4. あなたの配偶者は現在、所得が発生する経済活動に従事していますか。

① はい
② いいえ

M5. 入金, 月給, 社会保証または引退年金, 親戚から借りたお金などすべての所得を入れた場合、ご家庭の2016年世帯年収はいくらですか。税引き前収入でお答えください。

① 90万円未満 ② 90万円以上 180万円未満
③ 180万円以上 270万円未満 ④ 270万円以上 360万円未満
⑤ 360万円以上 450万円未満 ⑥ 450万円以上 540万円未満
⑦ 540万円以上 630万円未満 ⑧ 630万円以上 720万円未満
⑨ 720万円以上 810万円未満 ⑩ 810万円以上 900万円未満
⑪ 900万円以上

－ ご返答ありがとうございました －
