# Supplementary material for: The effect of maternal decisional authority on children's vaccination in East Asia
Source: PLoS One. 2018 Jul 12;13(7):e0200333. doi: 10.1371/journal.pone.0200333 (PMC6042723; doi:10.1371/journal.pone.0200333)
Supplement: S4 File — (PDF) [file pone.0200333.s004.pdf]

[설문지]

비약물적 중재와 백신 순응도에 영향을 미치는 커뮤니케이션 불평등 요인 분석 및 신종 감염병 대응체계의 개발 Ver 3.0

안녕하십니까?

본 조사는 한국연구재단의 지원을 받아 수행되는 『비약물적 중재와 백신순응도에 영향을 미치는 커뮤니케이션 불평등 요인』입니다.

이 연구는 백신접종에 영향을 미치는 사회맥락적 요인을 탐구하여 모성을 중심으로 백신접종률을 높이고 비약물적 중재를 강화하며 사회계층간 건강 커뮤니케이션의 불평등을 완화하기 위한 목적으로 실시되는 아시아권 국제비교 연구입니다. 설문지를 통해 수집된 응답내용은 「통계법」 제33조에 의거하여 완전히 무기명으로 처리되며 학술적 연구와 통계분석의 목적 이외의 용도로는 절대 사용되지 않습니다.

사실에 근거하여 귀하의 생각과 경험을 솔직하고 성의 있게 응답해 주시면 감사하겠습니다. 설문은 자율적으로 수행되는 것이므로 원하지 않으실 경우 조사에 참여하지 않거나 해당 문항에 응답하지 않으셔도 됩니다. 응답하신 설문자료는 국가의 학술적 연구조사에만 활용되며 분석결과는 전문 학술지에 게재됩니다.

귀하의 설문 응답은 우리나라를 포함하여 아시아권 주요 국가들의 백신접종률을 높이고 비약물적 중재 활동을 강화하여 전 지구적으로 증가하고 있는 신종감염병을 예방하고 조기 대응하는데 매우 귀중하게 사용될 것입니다.

감사합니다.

2017년 1월

동덕여자대학교 자연과학대학 보건관리학과
정민수 교수 (02-940-4483)

A. 모성에 대한 기초질문

A1. 귀하의 성별은 무엇입니까?

① 남성 -----> 설문조사 종료

② 여성

A2. 귀하의 연령은 어떻게 되십니까? 만 ( )세

[20~40세까지만 조사 참여 가능]

A2-1. 귀하의 거주 지역은 어디이십니까?

① 서울	⑦ 울산	⑬ 전남
② 인천	⑧ 경기	⑭ 경북
③ 대전	⑨ 강원	⑮ 경남
④ 광주	⑩ 충북	⑯ 제주
⑤ 부산	⑪ 충남	⑰ 세종
⑥ 대구	⑫ 전북	

A3. 귀하의 혼인상태는 어떻게 되십니까?

① 기혼(동거 중으로 사실혼 포함)

② 별거(이혼전제)

③ 사별/실종 -----> 설문조사 종료

④ 이혼 -----> 설문조사 종료

⑤ 미혼 -----> 설문조사 종료

A4. 귀하께서는 임신 경험이 있습니까?

① 예

② 아니오 -----> B1로 이동

A5. 귀하께서는 출산 경험이 있습니까?

① 예

② 아니오 -----> B1로 이동

[숫자만 입력 가능]

A5-1. 귀하께서 낳은 아들 또는 딸의 수는 총 몇 명입니까?

명

A5-2. 귀하의 자녀 중 미성년 자녀(19세 이하)가 있으십니까?

① 예

② 아니오

B. 미디어 활용

B1. 귀하께서는 **하루 평균** 아래의 미디어를 각각 얼마나 사용하십니까?

		전혀 사용하지 않음	10분 미만	10분 이상-30분 미만	30분 이상-60분 미만	1시간 이상 - 2시간 미만	2시간 이상 - 3시간 미만	3시간 이상 - 5시간 미만	5시간 이상
1	텔레비전 시청	①	②	③	④	⑤	⑥	⑦	⑧
2	라디오 청취	①	②	③	④	⑤	⑥	⑦	⑧
3	종이 신문 읽기	①	②	③	④	⑤	⑥	⑦	⑧
4	스마트폰으로 인터넷 검색	①	②	③	④	⑤	⑥	⑦	⑧
5	컴퓨터로 인터넷 검색	①	②	③	④	⑤	⑥	⑦	⑧

B2. 귀하는 평소 얼마나 적극적으로 **건강정보**를 찾는 편이십니까?

① 매우 열심히 찾는다　② 좀 열심히 찾는 편이다　③ 보통이다
④ 다소 멀리하는 편이다　⑤ 거의 찾지 않는다

B3. 귀하는 지난 **일주일** 동안 아래 매체를 통해 **본인이나 가족의 건강정보**를 찾은 적이 있습니까?

		전혀 찾지 않았다	1-2회	3-4회	5회 이상
1	텔레비전	①	②	③	④
2	라디오	①	②	③	④
3	종이 신문	①	②	③	④
4	도서	①	②	③	④
5	인터넷	①	②	③	④

B4. 사람들은 때때로 건강정보를 찾는데 어려움을 겪습니다. 귀하께서는 다음의 각 상황에서 얼마나 **어려움**을 경험하셨습니까?

		전혀 어려움이 없었다	약간 어려움이 있었다	매우 어려움이 있었다
1	정보가 너무 많았음	①	②	③
2	정보가 정확한 것인지 알 방법이 없었음	①	②	③
3	정보가 최신의 것인지 알 방법이 없었음	①	②	③
4	정보가 나의 상황과 관련된 것인지 알 수 없었음	①	②	③
5	찾아 본 정보가 전문적인 용어를 너무 많이 사용했음	①	②	③
6	온라인 탐색 도구나 소프트웨어 이용의 어려움	①	②	③
7	인터넷에 자유롭게 접근하기 어려움	①	②	③

C. 산전관리와 의료이용

C1. 귀하께서는 지난 **한 달** 동안 병원이나 의원에 몇 차례 **외래 진료**를 받았습니까?

① 지난 한 달 동안에 외래진료를 받은 적이 없다
② 1회 방문
③ 2회 방문
④ 3회 방문
⑤ 4회 방문
⑥ 5회 이상 방문 또는 입원

C2. 귀하께서는 지난 1년간 병·의원에서 치료나 검사를 받아 볼 필요가 있었으나, 받지 못한 적이 한 번이라도 있었습니까?

① 예 (받지 못한 적이 한 번이라도 있었다)
② 아니오 (받지 못한 적이 한 번도 없었다)

[A5에서 1인 사람만 응답, 그 외에는 C6로 이동]
C3. 귀하께서는 **가장 최근** 임신기간 동안 산전관리를 위해 몇 회의 의료이용을 받으셨습니까?

회

C4. **가장 최근** 출산 시 아이가 미숙아 혹은 조산아로 태어났습니까?

① 예
② 아니오

C5. **가장 최근** 출산 시 아이의 몸무게는 몇 킬로그램이었습니까?

	.		

KG

C6. 귀하의 키는 몇 센티미터입니까?

CM

C7. 귀하의 몸무게는 몇 킬로그램입니까?

		.	

KG

D. 백신접종

[A5-2에서 ①예(19세 이하의 자녀가 있는 경우)인 경우에만 응답, 그 외에는 D7로 이동]

[해당 상단 안내문은 D1~D6까지 모두 노출함]

<해당 질문은 귀하의 미성년 자녀(19세 이하)에게 해당하는 질문입니다. 미성년 자녀가 2명 이상인 경우, 가장 나이가 어린 자녀를 기준으로 응답해 주시기 바랍니다.>

D1. 결핵을 예방하기 위해 귀하의 자녀에게 **BCG 예방접종**을 실시하였습니까?

① 예

② 아니오

D2. 소아마비를 예방하기 위해 귀하의 자녀에게 **폴리오(Polio) 예방접종**을 실시하였습니까?

① 예

② 아니오

D3. 디프테리아, 파상풍, 백일해를 예방하기 위해 귀하의 자녀에게 **DPT 예방접종**을 실시하였습니까?

① 예

② 아니오

D4. 홍역, 볼거리, 풍진을 예방하기 위해 귀하의 자녀에게 **홍역, 볼거리, 풍진 예방접종**을 실시하였습니까?

① 예

② 아니오

D5. 일본뇌염을 예방하기 위해 귀하의 자녀에게 **일본뇌염 예방접종**을 실시하였습니까?

① 예

② 아니오

[D1~D5에서 모두 ② 아니오 선택 시 D7로 이동]

D6. 앞의 예방접종(결핵, 소아마비, 디프테리아, 파상풍, 백일해, 홍역, 볼거리, 풍진, 일본뇌염을 위해)을 자녀에게 실시하는데 있어 접종비용의 부담은 대체로 어떻게 하셨습니까?

① 국가에서 실시하는 무료 접종이었다

② 약간만 내가 직접 부담하였다

③ 절반 정도 내가 직접 부담하였다

④ 대부분 내가 직접 부담하였다

⑤ 개인적으로 가지고 있는 민간보험에서 부담하였다

D7. 귀하께서는 지난 1년 동안 독감 백신을 접종받은 적이 있습니까?(본인 접종)

① 예 ② 아니오

E. 건강수준 및 건강행태

E1. 귀하의 건강수준은 어떻습니까?

① 매우 나쁘다
② 나쁘다
③ 보통이다
④ 좋다
⑤ 매우 좋다

[A5에서 1인 사람만 응답, 그 외에는 E3로 이동]

<해당 질문은 귀하의 자녀에게 해당하는 질문입니다. 자녀가 2명 이상인 경우, 가장 나이가 어린 자녀를 기준으로 응답해 주시기 바랍니다.>

E2. 귀하의 자녀의 건강수준은 어떻습니까?

① 매우 나쁘다
② 나쁘다
③ 보통이다
④ 좋다
⑤ 매우 좋다

E3. 귀하께서는 현재 담배를 피우십니까?

① 예
② 아니오 -----> E4로 이동

E3-1. 귀하의 하루 평균 흡연량은 얼마입니까?

① 반 갑 이하 ② 한 갑 정도 ③ 한 갑 반 정도 ④ 두 갑 이상

E4. 귀하께서는 최근 1년간 1잔 이상의 술을 마신 적이 있으십니까?

① 예
② 아니오 -----> F1로 이동

E4-1. 귀하께서는 술을 얼마나 자주 마십니까?

① 한 달에 1번 미만
② 한 달에 1번 정도
③ 한 달에 2-4번 정도
④ 일주일에 2-3번 정도
⑤ 일주일에 4번 이상

E4-2. 귀하께서는 한 번에 술을 얼마나 마십니까? (소주, 맥주 구분 없이)

① 1-2잔 ② 3-4잔 ③ 5-6잔 ④ 7-9잔 이상

F. 위험 행태 및 자기효능감

F1. 귀하께서는 다음의 행동들을 얼마나 해보고 싶습니까?

		매우 그렇지 않다	그렇지 않다	그렇다	매우 그렇다
1	암벽 등반을 해보고 싶다	①	②	③	④
2	겁나더라도 스릴감 넘치는 것들을 해보고 싶다	①	②	③	④
3	수상스키나 서핑보드 같은 짜릿한 스포츠를 해보고 싶다	①	②	③	④
4	비행기에서 낙하산을 타고 뛰어내려보고 싶다	①	②	③	④
5	높은 산비탈에서 스키를 타고 빠르게 내려오고 싶다	①	②	③	④
6	번지점프를 해보고 싶다	①	②	③	④
7	놀이공원에서 청룡열차나 아찔한 놀이기구들을 타고 싶다	①	②	③	④
8	내 또래로 보이는 폭주족들과 어울려보고 싶다	①	②	③	④

F2. 다음의 각 문항에 대한 귀하의 생각을 보기 중에서 답해 주십시오.

		전혀 그렇지 않다	그렇지 않다	보통이다	그렇다	매우 그렇다
1	나는 자신감이 있다	①	②	③	④	⑤
2	나는 쉽게 포기 한다	①	②	③	④	⑤
3	나는 맡은바 일을 계획대로 수행할 수 있다	①	②	③	④	⑤
4	나는 어려운 일에 부딪히는 것을 피하는 편이다	①	②	③	④	⑤
5	어떤 일이 처음에 잘못 되었어도 끝까지 해 본다	①	②	③	④	⑤
6	나는 중요한 목표를 세우면 성취할 수 있다	①	②	③	④	⑤
7	나는 어떤 일이 복잡해 보이면 시도조차 안 한다	①	②	③	④	⑤
8	나는 뭔가 할 일이 있으면 바로 그 일을 시작 한다	①	②	③	④	⑤
9	예기치 못한 문제가 일어나면 나는 대처가 어렵다	①	②	③	④	⑤

G. 모성 역량

G1. 다음은 가족 내에서의 귀하의 의사결정에 대한 문항들입니다. 솔직하게 답변해 주십시오.

		응답자	응답자와 배우자가 (파트너) 함께	배우자 (파트너)	다른 누군가
1	귀하의 수입을 어떻게 사용할지에 대해 주로 누가 결정합니까?	①	②	③	④
2	귀하와 귀하의 배우자 중에서 누가 더 수입이 큽니까?	①	②	③	④
3	배우자의 수입을 어떻게 사용할지에 대해 주로 누가 결정합니까?	①	②	③	④
4	귀하가 의료이용을 하는 것에 대해 결정을 하는 사람은 누구입니까?	①	②	③	④
5	집 안의 물건을 구매하는 것은 주로 누가 결정합니까?	①	②	③	④
6	가족 또는 친척을 방문하는 것은 주로 누가 결정합니까?	①	②	③	④

G2. 다음은 삶에 대해 귀하께서 갖고 계신 주도적 역량을 묻는 문항들입니다. 솔직하게 답변해 주십시오.

		매우 그렇지 않다	그렇지 않다	보통 이다	그렇다	매우 그렇다
1	나는 내 삶에서 중요한 선택을 자유롭게 할 수 있다	①	②	③	④	⑤
2	나는 여성으로서 장점을 가지고 있다	①	②	③	④	⑤
3	나는 여자로서 나의 외모를 당당하게 표현한다	①	②	③	④	⑤
4	나는 나의 건강관리를 위한 시간을 확보하려고 노력한다	①	②	③	④	⑤
5	나는 나에게 맞는 운동을 꾸준히 실행한다	①	②	③	④	⑤
6	배우자에게 욕설이나 폭언을 들은 적이 있다	①	②	③	④	⑤
7	배우자와 말다툼을 하다가 손찌검을 당한 적이 있다	①	②	③	④	⑤
8	거절 의사를 밝혔음에도 배우자와 강제로 성관계를 맺어야 했던 적이 있다	①	②	③	④	⑤

H. 건강 문해력

H1. 다음은 귀하께서 건강에 대한 정보를 이해하고 활용하며 의사소통할 수 있는지 묻는 문항들입니다. 솔직하게 답하여 주십시오.

		문제없다	종종 어렵다	자주 어렵다
1	병원에서 제공하는 서식(예: 수술동의서, 진료안내서, 복약설명서)을 이해하고 쓸 수 있습니까?	①	②	③
2	환자 관리를 위한 차트의 내용을 이해하고 필요한 내용을 기입할 수 있습니까?	①	②	③
3	의사가 제공하는 의료정보가 담긴 인쇄물을 이해할 수 있습니까?	①	②	③
4	진료 예약을 하는 방법을 알고 있습니까?	①	②	③
5	모르는 건강 정보를 이해하기 위해서 의사에게 질문할 수 있습니까?	①	②	③

H2. 다음은 귀하의 **인터넷을 통한 건강정보 활용**에 관한 질문입니다. 솔직하게 답하여 주십시오.

		매우 그렇지 않다	그렇지 않다	보통 이다	그렇다	매우 그렇다
1	나는 인터넷에서 유용한 건강정보를 어디에서 찾을 수 있는지 알고 있다	①	②	③	④	⑤
2	나는 인터넷에서 찾은 건강정보를 어떻게 이용할지 알고 있다	①	②	③	④	⑤
3	나는 인터넷에서 찾은 건강정보가 양질의 것인지 구분할 수 있다	①	②	③	④	⑤
4	나는 건강과 관련한 의사결정을 위해 인터넷 건강정보를 활용하는 것에 자신이 있다	①	②	③	④	⑤

H3. 귀하께서는 평소에 보건 의료와 관련된 통계나 수치를 이해하는 것이 어렵습니까?

① 매우 쉽다 ② 쉽다 ③ 어렵다 ④ 매우 어렵다

H4. 다음 중 질병에 걸릴 위험이 가장 큰 것은 어느 것입니까?

① 100 분의 1 ② 1,000 분의 1 ③ 10 분의 1

H5. 사람들은 두 가지 말을 사용하여 사건이 발생할 확률에 대해 말합니다. 예를 들어, “가끔 발생한다”와 “5%의 가능성이 있다” 중에서 귀하는 어떤 방식의 표현을 선호합니까?

① 단어(가끔 발생한다)가 더 좋다

② 숫자(5%의 가능성이 있다)가 더 좋다

③ 어느 것이든 상관없다

I. 위험 지각

[A5에서 1인 사람만 응답, 그 외에는 I3로 이동]

I1. 다음은 귀하의 위험 지각에 대한 질문입니다. 질문의 상황이 **귀하의 자녀**에게 발생할 가능성이 어느 정도라고 생각하시는지 선택해 주십시오.

		매우 낮다	평균보다 낮다	평균이다	평균보다 높다	매우 높다
1	이번 겨울에 감기에 걸릴 가능성이 어느 정도라고 생각하십니까?	①	②	③	④	⑤
2	향후 1년 이내에 병으로 병원에 입원할 가능성이 어느 정도라고 생각하십니까?	①	②	③	④	⑤
3	향후 1년 이내에 뼈가 부러질 가능성이 어느 정도라고 생각하십니까?	①	②	③	④	⑤
4	향후 1년 이내에 수두에 걸릴 가능성이 어느 정도라고 생각하십니까?	①	②	③	④	⑤
5	향후 1년 이내에 고열에 시달릴 가능성이 어느 정도라고 생각하십니까?	①	②	③	④	⑤

I2. 질문의 상황이 귀하의 자녀가 아닌 **다른 아이들**에게 발생할 가능성이 어느 정도라고 생각하시는지 선택해 주십시오.

		매우 낮다	평균보다 낮다	평균이다	평균보다 높다	매우 높다
1	이번 겨울에 감기에 걸릴 가능성이 어느 정도라고 생각하십니까?	①	②	③	④	⑤
2	향후 1년 이내에 병으로 병원에 입원할 가능성이 어느 정도라고 생각하십니까?	①	②	③	④	⑤
3	향후 1년 이내에 뼈가 부러질 가능성이 어느 정도라고 생각하십니까?	①	②	③	④	⑤
4	향후 1년 이내에 수두에 걸릴 가능성이 어느 정도라고 생각하십니까?	①	②	③	④	⑤
5	향후 1년 이내에 고열에 시달릴 가능성이 어느 정도라고 생각하십니까?	①	②	③	④	⑤

I3. 아래 문항들을 읽고 해당하는 곳에 선택해 주십시오.

		전혀 없다	매우 낮다	일반인과 비슷하다	다소 높다	매우 높다
1	귀하께서 1년 이내에 유행성 전염병에 걸릴 가능성은 어느 정도라고 보십니까?	①	②	③	④	⑤
2	귀하께서 1년 이내에 암에 걸릴 가능성은 어느 정도라고 보십니까?	①	②	③	④	⑤
3	귀하께서 1년 이내에 현재의 남편과 이혼할 가능성은 어느 정도라고 보십니까?	①	②	③	④	⑤

J. 비약물적 중재

J1. 다음은 WHO에서 제시한 올바른 손 씻기입니다. 귀하께서는 평소에 이러한 손 씻기 규칙을 지키십니까?

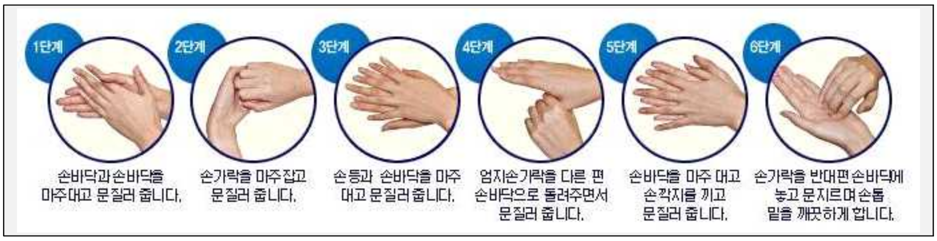

① 거의 지키지 않는다
② 지키지 않는 편이다
③ 보통이다
④ 지키는 편이다
⑤ 매우 잘 지킨다

J2. 귀하는 평소 생활에서 다음과 같은 질병 예방 수칙을 실천하십니까?

		전혀 실천하지 않는다	거의 실천하지 않는다	보통이다	가급적 실천한다	반드시 실천한다
1	손 씻기 혹은 손 세정제 사용하기	①	②	③	④	⑤
2	전염병에 감염된 사람과의 접촉을 피하기	①	②	③	④	⑤
3	눈, 코, 입 등을 손으로 만지지 않기	①	②	③	④	⑤
4	전염병 유행 시 외출 자제하기	①	②	③	④	⑤
5	사람들과의 접촉이 빈번한 장소 피하기	①	②	③	④	⑤
6	마스크 착용하기	①	②	③	④	⑤

J3. 귀하는 아래의 정보원들 각각에 대해서 얼마나 신뢰하십니까?

		전혀 신뢰하지 않는다	신뢰하지 않는다	다소 신뢰한다	매우 신뢰한다
1	텔레비전	①	②	③	④
2	라디오	①	②	③	④
3	종이 신문	①	②	③	④
4	인터넷	①	②	③	④
5	정부 당국(보건복지부, 질병관리본부)	①	②	③	④
6	의사 또는 보건 전문가	①	②	③	④
7	해당 질환에 걸려본 가족이나 지인	①	②	③	④

K. 건강관련 지식

K1. 다음은 에이즈에 관한 질문입니다. 각 문항이 맞는지 틀린지 귀하의 생각을 선택해 주십시오.

		맞다	틀리다
1	에이즈 감염인과 같은 물 잔을 사용하면 에이즈에 감염될 수 있다	①	②
2	에이즈 감염인과 식사를 같이 하면 에이즈에 감염될 수 있다	①	②
3	에이즈는 모기를 통해서 감염될 수 있다	①	②
4	에이즈는 성관계시 콘돔을 사용하면 예방할 수 있다	①	②
5	에이즈는 적절한 치료와 건강관리를 하면 30년 이상 살 수 있다	①	②

K2. 우리나라 성인의 건강에 대한 지식 수준이 평균 50점이라고 할 경우 귀하의 건강지식 점수는 몇 점이라고 생각하십니까? 해당 영역에 선택해 주십시오.(10점 단위)

0점	10점	20점	30점	40점	국민 평균 50점	60점	70점	80점	90점	100점
1	2	3	4	5	6	7	8	9	10	11

K3. 다음은 신종 감염병에 대한 인식도에 관한 질문입니다. 해당 영역에 선택해 주십시오.

		전혀 그렇지 않다	그렇지 않다	보통 이다	그렇다	매우 그렇다
1	귀하는 신종 감염병 대유행을 명확히 설명하실 수 있습니까?	①	②	③	④	⑤
2	귀하는 최근 감염병 대유행 시 뉴스 등을 통해 진행과정에 계속적인 관심을 가졌습니까?	①	②	③	④	⑤
3	귀하는 앞으로도 우리나라에 신종 감염병으로 인한 대유행의 가능성이 높다고 생각하십니까?	①	②	③	④	⑤
4	귀하는 최근 감염병 대유행에 대한 WHO(세계보건기구)의 대응이 적절하였다고 생각하십니까?	①	②	③	④	⑤
5	귀하는 최근 감염병 대유행에 대한 정부의 대응이 적절하였다고 생각하십니까?	①	②	③	④	⑤

K4. 다음의 신종 인플루엔자에 관하여 귀하의 생각을 선택해 주십시오.

		그렇다	그렇지 않다
1	신종 인플루엔자의 병원체는 세균이 아닌 바이러스이다	①	②
2	신종 인플루엔자는 예방접종을 통해 예방이 가능하다	①	②
3	신종 인플루엔자에 걸리면 발열(37.8℃), 콧물, 인후통, 기침과 같은 증상이 나타난다	①	②

4	신종 인플루엔자는 환자의 침과 같은 비말(침방울)에 의해 주로 전파된다	①	②
5	신종 인플루엔자에 걸렸는데 치료하지 않으면 대부분 사망한다	①	②
6	손을 자주 씻으면 신종인플루엔자를 예방하는데 큰 도움이 된다	①	②

K5. 다음의 메르스(중동호흡기증후군, MERS)에 관하여 귀하의 생각을 선택해 주십시오.

		그렇다	그렇지 않다
1	메르스는 증상 없는 잠복기 중에도 전파된다	①	②
2	메르스는 보호장비 없이 환자를 돌보는 등의 행동을 하지 않았다면 사람 간에 쉽게 전파되지 않는다	①	②
3	메르스의 주요 증상으로는 발열, 기침, 호흡곤란 등이 있다	①	②
4	환자가 이미 다녀갔던 병원에 방문하여도 메르스에 전염될 수 있다	①	②
5	증상이 발생한 환자와 밀접하게 접촉한 사람은 최종일로부터 7일간 자택이나 지정된 의료기관에 격리된다	①	②
6	사람이 많이 붐비는 장소의 방문을 자제하는 것은 메르스를 예방하는데 도움이 된다	①	②
7	귀국 후 14일 이내 발열, 호흡곤란 등 호흡기 이상증세가 있을 경우 곧바로 보건소 등에 신고해야한다	①	②

K6. 다음의 지카 바이러스(Zika virus)에 관하여 귀하의 생각을 선택해 주십시오.

		그렇다	그렇지 않다
1	지카 바이러스는 흰줄숲모기로 전파 가능하다	①	②
2	지카 바이러스는 헌혈로 추가 전파되지 않는다	①	②
3	의심환자의 경우 가임 여성은 지카 바이러스 발생 국가로부터 귀국 후 최소 2개월간 임신을 연기해야 한다	①	②
4	지카 바이러스는 감염되면 사망에 이를 수 있다	①	②
5	지카 바이러스는 침과 같은 비말(침방울)에 의해 주로 전파된다	①	②
6	지카 바이러스에 감염될 경우 최대 2주 이내에 발진, 발열, 관절통, 근육통, 눈 충혈과 같은 증상이 나타난다	①	②
7	임신부가 (특히 임신 초기에) 감염될 경우 태아 소두증을 유발할 수 있다	①	②

L. 건강관련 삶의 질과 사회적 지지

L1. 다음은 귀하의 건강 상태에 관한 질문입니다. 해당하는 곳에 선택해 주십시오.

		매우 그렇다	약간 그렇다	그렇지 않다
1	나는 걷는데 어려움이 없다	①	②	③
2	나는 목욕을 하거나 옷을 입는데 지장이 없다	①	②	③
3	나는 일상 활동을 하는데 지장이 없다	①	②	③
4	나는 통증이나 불편감이 없다	①	②	③
5	나는 불안하거나 우울하지 않다	①	②	③

L2. 아래 문항들을 읽고 해당하는 곳에 선택해 주십시오.

		항상 있었다	거의 언제나 있었다	종종 여러번 있었다	간혹 있었다	전혀 없었다
1	지난 한 달 동안 살아가는데 정신적, 신체적으로 감당하기 힘들다고 느끼신 적이 있습니까?	①	②	③	④	⑤
2	지난 한 달 동안 자신의 생활신념에 따라 살려고 애쓰시다가 좌절을 느끼신 적이 있습니까?	①	②	③	④	⑤
3	지난 한 달 동안 한 인간으로서 기본적인 욕구가 충족되지 않았다고 느끼신 적이 있습니까?	①	②	③	④	⑤
4	지난 한 달 동안 미래에 대해 불확실하게 느끼거나 불안해하신 적이 있습니까?	①	②	③	④	⑤
5	지난 한 달 동안 할 일이 너무 많아 정말 중요한 일들을 잊으신 적이 있습니까?	①	②	③	④	⑤

L3. 귀하께서는 다음 문장에 대해 얼마나 동의하십니까?

		매우 그렇지 않다	다소 그렇지 않다	다소 그렇다	매우 그렇다
1	주변 사람들은 이웃을 돕는데 적극적이다	①	②	③	④
2	이웃 사람들은 믿을 수 있다	①	②	③	④
3	이웃 사람들을 자주 만나고 모인다	①	②	③	④

L4. 귀하께서는 가족 이외의 친한 친구를 얼마나 자주 만납니까?

① 한 달에 1번 미만 ② 한 달에 1번 ③ 한 달에 2-3번 ④ 주 1회
⑤ 주 2-3회 ⑥ 거의 매일

L5. 귀하께서는 일주일에 얼마나 자주 혼자서 밥을 드십니까?

① 거의 매일 ② 일주일에 5회 이상 ③ 일주일에 3-4회 ④ 일주일에 1-2회
⑤ 전혀 혼자 먹지 않는다

M. 응답자의 일반적 특성

M1. 귀하의 최종학력은 어떻게 되십니까?
① 초등학교 졸업 이하
② 중학교 졸업
③ 고등학교 졸업
④ 대학교 졸업
⑤ 대학원 재학 이상

M2. 귀하께서는 현재 소득이 발생하는 경제활동에 종사하고 계십니까?
① 예
② 아니오 -----> M3로 이동

M2-1. 귀하의 종사상 지위는 무엇입니까?
① 상시근로자
② 임시근로자

M3. 귀하의 배우자의 최종학력은 어떻게 되십니까?
① 초등학교 졸업 이하
② 중학교 졸업
③ 고등학교 졸업
④ 대학교 졸업
⑤ 대학원 재학 이상

M4. 귀하의 배우자께서는 현재 소득이 발생하는 경제활동에 종사하고 계십니까?
① 예
② 아니오

M5. 임금, 월급, 사회보장 또는 은퇴 연금, 친척의 도움 등 모든 소득을 포함하여 **귀 가정(부부합산)**의 2016년 **총합계 수입(연봉)**은 얼마입니까? 세전 수입으로 말씀해 주십시오.
① 1,000만원 미만
② 1,000만원 이상 ~ 2,000만원 미만
③ 2,000만원 이상 ~ 3,000만원 미만
④ 3,000만원 이상 ~ 4,000만원 미만
⑤ 4,000만원 이상 ~ 5,000만원 미만
⑥ 5,000만원 이상 ~ 6,000만원 미만
⑦ 6,000만원 이상 ~ 7,000만원 미만
⑧ 7,000만원 이상 ~ 8,000만원 미만
⑨ 8,000만원 이상 ~ 9,000만원 미만
⑩ 9,000만원 이상 ~ 1억 미만
⑪ 1억 이상

\- 설문에 응해주셔서 대단히 감사합니다! -
