# Supplementary material for: The effect of maternal decisional authority on children's vaccination in East Asia
Source: PLoS One. 2018 Jul 12;13(7):e0200333. doi: 10.1371/journal.pone.0200333 (PMC6042723; doi:10.1371/journal.pone.0200333)
Supplement: S1 Table — (PDF) [file pone.0200333.s005.pdf]

```
CROSSTABS
  /TABLES=B3_ca3 B3_2_ca3 B3_3_ca3 B3_4_ca3 B3_5_ca3 BY C
  /FORMAT=AVALUE TABLES
  /STATISTICS=CHISQ
  /CELLS=COUNT ROW
  /COUNT ROUND CELL.
```

Crosstabs

Case Processing Summary

	Cases					
	Valid		Missing		Total	
	N	Percent	N	Percent	N	Percent
지난 일주일간 텔레비전으로 건강정보 찾았는지 * C	1571	100.0%	0	0.0%	1571	100.0%
지난 일주일간 라디오로 건강정보 찾았는지 * C	1571	100.0%	0	0.0%	1571	100.0%
지난 일주일간 종이신문으로 건강정보 찾았는지 * C	1571	100.0%	0	0.0%	1571	100.0%
지난 일주일간 도서로 건강정보 찾았는지 * C	1571	100.0%	0	0.0%	1571	100.0%
지난 일주일간 인터넷으로 건강정보 찾았는지 * C	1571	100.0%	0	0.0%	1571	100.0%

지난 일주일간 텔레비전으로 건강정보 찾았는지 * C

Crosstab

			C			Total
			한국	중국	일본	
지난 일주일간 텔레비전으로 건강정보 찾았는지	(1) 전혀 찾지 않았다	Count	262	150	319	731
		% within 지난 일주일간 텔레비전으로 건강정보 찾았는지	35.8%	20.5%	43.6%	100.0%
	(2) 1-2회	Count	253	247	89	589
		% within 지난 일주일간 텔레비전으로 건강정보 찾았는지	43.0%	41.9%	15.1%	100.0%
	(3) 3회 이상	Count	59	169	23	251
		% within 지난 일주일간 텔레비전으로 건강정보 찾았는지	23.5%	67.3%	9.2%	100.0%
Total		Count	574	566	431	1571
		% within 지난 일주일간 텔레비전으로 건강정보 찾았는지	36.5%	36.0%	27.4%	100.0%

Chi-Square Tests

	Value	df	Asymptotic Significance (2-sided)
Pearson Chi-Square	274.284[a]	4	.000
Likelihood Ratio	276.752	4	.000
Linear-by-Linear Association	35.401	1	.000
N of Valid Cases	1571		

a. 0 cells (.0%) have expected count less than 5. The minimum expected count is 68.86.

지난 일주일간 라디오로 건강정보 찾았는지 * C

Crosstab

			C			Total
			한국	중국	일본	
지난 일주일간 라디오로 건강정보 찾았는지	(1) 전혀 찾지 않았다	Count	513	307	419	1239
		% within 지난 일주일간 라디오로 건강정보 찾았는지	41.4%	24.8%	33.8%	100.0%
	(2) 1-2회	Count	54	190	7	251
		% within 지난 일주일간 라디오로 건강정보 찾았는지	21.5%	75.7%	2.8%	100.0%
	(3) 3회 이상	Count	7	69	5	81
		% within 지난 일주일간 라디오로 건강정보 찾았는지	8.6%	85.2%	6.2%	100.0%
Total		Count	574	566	431	1571
		% within 지난 일주일간 라디오로 건강정보 찾았는지	36.5%	36.0%	27.4%	100.0%

Chi-Square Tests

	Value	df	Asymptotic Significance (2-sided)
Pearson Chi-Square	335.592[a]	4	.000
Likelihood Ratio	350.322	4	.000
Linear-by-Linear Association	.610	1	.435
N of Valid Cases	1571		

a. 0 cells (.0%) have expected count less than 5. The minimum expected count is 22.22.

지난 일주일간 종이신문으로 건강정보 찾았는지 * C

Crosstab

			C			Total
			한국	중국	일본	
지난 일주일간 종이신문으로 건강정보 찾았는지	(1) 전혀 찾지 않았다	Count	502	223	401	1126
		% within 지난 일주일간 종이신문으로 건강정보 찾았는지	44.6%	19.8%	35.6%	100.0%
	(2) 1-2회	Count	58	225	28	311
		% within 지난 일주일간 종이신문으로 건강정보 찾았는지	18.6%	72.3%	9.0%	100.0%
	(3) 3회 이상	Count	14	118	2	134
		% within 지난 일주일간 종이신문으로 건강정보 찾았는지	10.4%	88.1%	1.5%	100.0%
Total		Count	574	566	431	1571
		% within 지난 일주일간 종이신문으로 건강정보 찾았는지	36.5%	36.0%	27.4%	100.0%

Chi-Square Tests

	Value	df	Asymptotic Significance (2-sided)
Pearson Chi-Square	467.796[a]	4	.000
Likelihood Ratio	479.708	4	.000
Linear-by-Linear Association	.004	1	.948
N of Valid Cases	1571		

a. 0 cells (.0%) have expected count less than 5. The minimum expected count is 36.76.

지난 일주일간 도서로 건강정보 찾았는지 * C

Crosstab

			C			Total
			한국	중국	일본	
지난 일주일간 도서로 건강정보 찾았는지	(1) 전혀 찾지 않았다	Count	382	163	385	930
		% within 지난 일주일간 도서로 건강정보 찾았는지	41.1%	17.5%	41.4%	100.0%
	(2) 1-2회	Count	156	192	38	386
		% within 지난 일주일간 도서로 건강정보 찾았는지	40.4%	49.7%	9.8%	100.0%
	(3) 3회 이상	Count	36	211	8	255
		% within 지난 일주일간 도서로 건강정보 찾았는지	14.1%	82.7%	3.1%	100.0%
Total		Count	574	566	431	1571
		% within 지난 일주일간 도서로 건강정보 찾았는지	36.5%	36.0%	27.4%	100.0%

Chi-Square Tests

	Value	df	Asymptotic Significance (2-sided)
Pearson Chi-Square	469.385[a]	4	.000
Likelihood Ratio	496.481	4	.000
Linear-by-Linear Association	15.112	1	.000
N of Valid Cases	1571		

a. 0 cells (.0%) have expected count less than 5. The minimum expected count is 69.96.

지난 일주일간 인터넷으로 건강정보 찾았는지 * C

Crosstab

			C			Total
			한국	중국	일본	
지난 일주일간 인터넷으로 건강정보 찾았는지	(1) 전혀 찾지 않았다	Count	34	34	163	231
		% within 지난 일주일간 인터넷으로 건강정보 찾았는지	14.7%	14.7%	70.6%	100.0%
	(2) 1-2회	Count	207	167	160	534
		% within 지난 일주일간 인터넷으로 건강정보 찾았는지	38.8%	31.3%	30.0%	100.0%
	(3) 3회 이상	Count	333	365	108	806
		% within 지난 일주일간 인터넷으로 건강정보 찾았는지	41.3%	45.3%	13.4%	100.0%
Total		Count	574	566	431	1571
		% within 지난 일주일간 인터넷으로 건강정보 찾았는지	36.5%	36.0%	27.4%	100.0%

Chi-Square Tests

	Value	df	Asymptotic Significance (2-sided)
Pearson Chi-Square	303.226[a]	4	.000
Likelihood Ratio	284.703	4	.000
Linear-by-Linear Association	174.247	1	.000
N of Valid Cases	1571		

a. 0 cells (.0%) have expected count less than 5. The minimum expected count is 63.37.

```
LOGISTIC REGRESSION VARIABLES Dep_4_bi
  /METHOD=ENTER M1_ca3 M5_ca7 F_HL F_ISB F_SE B3_ca3 B3_2_ca3 B3_3_ca3 B3_4_ca3 B3_5_c
a3 F_MC1
    F_MC2
  /PRINT=CI(95)
  /CRITERIA=PIN(0.05) POUT(0.10) ITERATE(20) CUT(0.5).
```

Logistic Regression

Case Processing Summary

Unweighted Cases[a]		N	Percent
Selected Cases	Included in Analysis	1496	95.2
	Missing Cases	75	4.8
	Total	1571	100.0
Unselected Cases		0	.0
Total		1571	100.0

a. If weight is in effect, see classification table for the total number of cases.

Dependent Variable Encoding

Original Value	Internal Value
1가지 이상 미접종	0
모두 접종	1

Block 0: Beginning Block

Classification Table[a,b]

Observed			Predicted: 4가지 국가백신 접종여부 – 1가지 이상 미접종	Predicted: 4가지 국가백신 접종여부 – 모두 접종	Predicted: Percentage Correct
Step 0	4가지 국가백신 접종여부	1가지 이상 미접종	0	260	.0
		모두 접종	0	1236	100.0
	Overall Percentage				82.6

a. Constant is included in the model.

b. The cut value is .500

Variables in the Equation

		B	S.E.	Wald	df	Sig.	Exp(B)
Step 0	Constant	1.559	.068	522.068	1	.000	4.754

Variables not in the Equation

			Score	df	Sig.
Step 0	Variables	M1_ca3	1.572	1	.210
		M5_ca7	.088	1	.767
		F_HL	10.389	1	.001
		F_ISB	.027	1	.870
		F_SE	4.142	1	.042
		B3_ca3	.310	1	.577
		B3_2_ca3	3.665	1	.056
		B3_3_ca3	.551	1	.458
		B3_4_ca3	.083	1	.773
		B3_5_ca3	.707	1	.400
		F_MC1	10.637	1	.001
		F_MC2	.197	1	.657
	Overall Statistics		37.849	12	.000

Block 1: Method = Enter

Omnibus Tests of Model Coefficients

		Chi-square	df	Sig.
Step 1	Step	38.025	12	.000
	Block	38.025	12	.000
	Model	38.025	12	.000

Model Summary

Step	-2 Log likelihood	Cox & Snell R Square	Nagelkerke R Square
1	1343.847[a]	.025	.042

a. Estimation terminated at iteration number 5 because parameter estimates changed by less than .001.

Classification Table[a]

Observed			Predicted: 4가지 국가백신 접종여부 — 1가지 이상 미접종	Predicted: 4가지 국가백신 접종여부 — 모두 접종	Predicted: Percentage Correct
Step 1	4가지 국가백신 접종여부	1가지 이상 미접종	0	260	.0
		모두 접종	0	1236	100.0
	Overall Percentage				82.6

a. The cut value is .500

Variables in the Equation

		B	S.E.	Wald	df	Sig.	Exp(B)	95% C.I.for EXP(B)	
								Lower	Upper
Step 1[a]	M1_ca3	.146	.155	.880	1	.348	1.157	.853	1.568
	M5_ca7	-.012	.033	.129	1	.719	.988	.927	1.053
	F_HL	.183	.067	7.362	1	.007	1.201	1.052	1.370
	F_ISB	-.420	.370	1.289	1	.256	.657	.318	1.357
	F_SE	.214	.089	5.821	1	.016	1.239	1.041	1.475
	B3_ca3	.209	.188	1.239	1	.266	1.232	.853	1.779
	B3_2_ca3	.586	.243	5.802	1	.016	1.797	1.115	2.894
	B3_3_ca3	.052	.213	.059	1	.809	1.053	.694	1.598
	B3_4_ca3	.044	.203	.048	1	.827	1.045	.702	1.557
	B3_5_ca3	-.003	.203	.000	1	.990	.997	.670	1.484
	F_MC1	.232	.073	10.174	1	.001	1.262	1.094	1.455
	F_MC2	-.275	.093	8.766	1	.003	.760	.634	.911
	Constant	.159	1.223	.017	1	.896	1.173		

a. Variable(s) entered on step 1: M1_ca3, M5_ca7, F_HL, F_ISB, F_SE, B3_ca3, B3_2_ca3, B3_3_ca3, B3_4_ca3, B3_5_ca3, F_MC1, F_MC2.

```
LOGISTIC REGRESSION VARIABLES Dep_4_bi
  /METHOD=ENTER M1_ca3 M5_ca7 F_SE F_HL B3_ca3 B3_2_ca3 B3_3_ca3 B3_4_ca3 B3_5_ca3 F_M
C1 F_MC2
  /PRINT=CI(95)
  /CRITERIA=PIN(0.05) POUT(0.10) ITERATE(20) CUT(0.5).
```

Logistic Regression

Case Processing Summary

Unweighted Cases[a]		N	Percent
Selected Cases	Included in Analysis	1496	95.2
	Missing Cases	75	4.8
	Total	1571	100.0
Unselected Cases		0	.0
Total		1571	100.0

a. If weight is in effect, see classification table for the total number of cases.

Dependent Variable Encoding

Original Value	Internal Value
1가지 이상 미접종	0
모두 접종	1

Block 0: Beginning Block

Classification Table[a,b]

Observed			Predicted: 4가지 국가백신 접종여부: 1가지 이상 미접종	모두 접종	Percentage Correct
Step 0	4가지 국가백신 접종여부	1가지 이상 미접종	0	260	.0
		모두 접종	0	1236	100.0
	Overall Percentage				82.6

a. Constant is included in the model.

b. The cut value is .500

Variables in the Equation

		B	S.E.	Wald	df	Sig.	Exp(B)
Step 0	Constant	1.559	.068	522.068	1	.000	4.754

Variables not in the Equation

			Score	df	Sig.
Step 0	Variables	M1_ca3	1.572	1	.210
		M5_ca7	.088	1	.767
		F_SE	4.142	1	.042
		F_HL	10.389	1	.001
		B3_ca3	.310	1	.577
		B3_2_ca3	3.665	1	.056
		B3_3_ca3	.551	1	.458
		B3_4_ca3	.083	1	.773
		B3_5_ca3	.707	1	.400
		F_MC1	10.637	1	.001
		F_MC2	.197	1	.657
	Overall Statistics		36.686	11	.000

Block 1: Method = Enter

Omnibus Tests of Model Coefficients

		Chi-square	df	Sig.
Step 1	Step	36.759	11	.000
	Block	36.759	11	.000
	Model	36.759	11	.000

Model Summary

Step	-2 Log likelihood	Cox & Snell R Square	Nagelkerke R Square
1	1345.114[a]	.024	.040

a. Estimation terminated at iteration number 5 because parameter estimates changed by less than .001.

Classification Table[a]

Observed			Predicted		
			4가지 국가백신 접종여부		Percentage Correct
			1가지 이상 미접종	모두 접종	
Step 1	4가지 국가백신 접종여부	1가지 이상 미접종	0	260	.0
		모두 접종	0	1236	100.0
	Overall Percentage				82.6

a. The cut value is .500

Variables in the Equation

		B	S.E.	Wald	df	Sig.	Exp(B)	95% C.I.for EXP(B)	
								Lower	Upper
Step 1[a]	M1_ca3	.147	.155	.899	1	.343	1.159	.855	1.571
	M5_ca7	-.013	.033	.154	1	.695	.987	.926	1.052
	F_SE	.216	.089	5.916	1	.015	1.242	1.043	1.478
	F_HL	.185	.067	7.512	1	.006	1.203	1.054	1.373
	B3_ca3	.053	.127	.173	1	.678	1.054	.822	1.352
	B3_2_ca3	.456	.213	4.598	1	.032	1.577	1.040	2.393
	B3_3_ca3	-.076	.180	.180	1	.672	.926	.651	1.319
	B3_4_ca3	-.119	.143	.701	1	.402	.887	.671	1.173
	B3_5_ca3	-.189	.118	2.579	1	.108	.828	.657	1.043
	F_MC1	.235	.073	10.447	1	.001	1.264	1.097	1.458
	F_MC2	-.278	.093	8.994	1	.003	.757	.631	.908
	Constant	1.462	.412	12.568	1	.000	4.313		

a. Variable(s) entered on step 1: M1_ca3, M5_ca7, F_SE, F_HL, B3_ca3, B3_2_ca3, B3_3_ca3, B3_4_ca3, B3_5_ca3, F_MC1, F_MC2.

```
LOGISTIC REGRESSION VARIABLES Dep_4_bi
  /METHOD=ENTER M1_ca3 M5_ca7 F_SE F_HL B3_ca3 B3_2_ca3 B3_3_ca3 B3_4_ca3 B3_5_ca3
  /PRINT=CI(95)
  /CRITERIA=PIN(0.05) POUT(0.10) ITERATE(20) CUT(0.5).
```

Logistic Regression

Case Processing Summary

Unweighted Cases[a]		N	Percent
Selected Cases	Included in Analysis	1519	96.7
	Missing Cases	52	3.3
	Total	1571	100.0
Unselected Cases		0	.0
Total		1571	100.0

a. If weight is in effect, see classification table for the total number of cases.

Dependent Variable Encoding

Original Value	Internal Value
1가지 이상 미접종	0
모두 접종	1

Block 0: Beginning Block

Classification Table[a,b]

Observed			Predicted: 4가지 국가백신 접종여부: 1가지 이상 미접종	Predicted: 4가지 국가백신 접종여부: 모두 접종	Percentage Correct
Step 0	4가지 국가백신 접종여부	1가지 이상 미접종	0	266	.0
		모두 접종	0	1253	100.0
	Overall Percentage				82.5

a. Constant is included in the model.

b. The cut value is .500

Variables in the Equation

		B	S.E.	Wald	df	Sig.	Exp(B)
Step 0	Constant	1.550	.068	527.019	1	.000	4.711

Variables not in the Equation

			Score	df	Sig.
Step 0	Variables	M1_ca3	2.121	1	.145
		M5_ca7	.029	1	.865
		F_SE	3.414	1	.065
		F_HL	11.149	1	.001
		B3_ca3	.231	1	.631
		B3_2_ca3	3.131	1	.077
		B3_3_ca3	.352	1	.553
		B3_4_ca3	.001	1	.979
		B3_5_ca3	1.030	1	.310
	Overall Statistics		22.031	9	.009

Block 1: Method = Enter

Omnibus Tests of Model Coefficients

		Chi-square	df	Sig.
Step 1	Step	21.684	9	.010
	Block	21.684	9	.010
	Model	21.684	9	.010

Model Summary

Step	-2 Log likelihood	Cox & Snell R Square	Nagelkerke R Square
1	1387.660[a]	.014	.023

a. Estimation terminated at iteration number 4 because parameter estimates changed by less than .001.

Classification Table[a]

Observed			Predicted		
			4가지 국가백신 접종여부		Percentage Correct
			1가지 이상 미접종	모두 접종	
Step 1	4가지 국가백신 접종여부	1가지 이상 미접종	0	266	.0
		모두 접종	0	1253	100.0
	Overall Percentage				82.5

a. The cut value is .500

Variables in the Equation

		B	S.E.	Wald	df	Sig.	Exp(B)	95% C.I.for EXP(B)	
								Lower	Upper
Step 1[a]	M1_ca3	.151	.153	.984	1	.321	1.164	.863	1.570
	M5_ca7	-.016	.032	.247	1	.619	.984	.924	1.048
	F_SE	.112	.077	2.129	1	.145	1.118	.962	1.300
	F_HL	.196	.065	9.137	1	.003	1.217	1.071	1.382
	B3_ca3	.027	.125	.047	1	.828	1.027	.805	1.312
	B3_2_ca3	.418	.207	4.087	1	.043	1.518	1.013	2.276
	B3_3_ca3	-.070	.175	.158	1	.691	.933	.662	1.314
	B3_4_ca3	-.158	.138	1.308	1	.253	.854	.652	1.119
	B3_5_ca3	-.171	.117	2.135	1	.144	.843	.670	1.060
	Constant	1.535	.407	14.213	1	.000	4.639		

a. Variable(s) entered on step 1: M1_ca3, M5_ca7, F_SE, F_HL, B3_ca3, B3_2_ca3, B3_3_ca3, B3_4_ca3, B3_5_ca3.

```
LOGISTIC REGRESSION VARIABLES Dep_4_bi
  /METHOD=ENTER M1_ca3 M5_ca7 F_SE F_HL
  /PRINT=CI(95)
  /CRITERIA=PIN(0.05) POUT(0.10) ITERATE(20) CUT(0.5).
```

Logistic Regression

Case Processing Summary

Unweighted Cases[a]		N	Percent
Selected Cases	Included in Analysis	1519	96.7
	Missing Cases	52	3.3
	Total	1571	100.0
Unselected Cases		0	.0
Total		1571	100.0

a. If weight is in effect, see classification table for the total number of cases.

Dependent Variable Encoding

Original Value	Internal Value
1가지 이상 미접종	0
모두 접종	1

Block 0: Beginning Block

Classification Table[a,b]

Observed			Predicted: 4가지 국가백신 접종여부 1가지 이상 미접종	Predicted: 4가지 국가백신 접종여부 모두 접종	Percentage Correct
Step 0	4가지 국가백신 접종여부	1가지 이상 미접종	0	266	.0
		모두 접종	0	1253	100.0
	Overall Percentage				82.5

a. Constant is included in the model.

b. The cut value is .500

Variables in the Equation

		B	S.E.	Wald	df	Sig.	Exp(B)
Step 0	Constant	1.550	.068	527.019	1	.000	4.711

Variables not in the Equation

			Score	df	Sig.
Step 0	Variables	M1_ca3	2.121	1	.145
		M5_ca7	.029	1	.865
		F_SE	3.414	1	.065
		F_HL	11.149	1	.001
	Overall Statistics		13.828	4	.008

Block 1: Method = Enter

Omnibus Tests of Model Coefficients

		Chi-square	df	Sig.
Step 1	Step	13.098	4	.011
	Block	13.098	4	.011
	Model	13.098	4	.011

Model Summary

Step	-2 Log likelihood	Cox & Snell R Square	Nagelkerke R Square
1	1396.246[a]	.009	.014

a. Estimation terminated at iteration number 4 because parameter estimates changed by less than .001.

Classification Table[a]

Observed			Predicted: 4가지 국가백신 접종여부 — 1가지 이상 미접종	Predicted: 4가지 국가백신 접종여부 — 모두 접종	Percentage Correct
Step 1	4가지 국가백신 접종여부	1가지 이상 미접종	0	266	.0
		모두 접종	0	1253	100.0
	Overall Percentage				82.5

a. The cut value is .500

Variables in the Equation

		B	S.E.	Wald	df	Sig.	Exp(B)	95% C.I.for EXP(B) Lower	95% C.I.for EXP(B) Upper
Step 1[a]	M1_ca3	.132	.151	.771	1	.380	1.142	.849	1.535
	M5_ca7	-.005	.032	.029	1	.865	.995	.935	1.058
	F_SE	.080	.069	1.369	1	.242	1.084	.947	1.240
	F_HL	.191	.064	8.834	1	.003	1.210	1.067	1.373
	Constant	1.337	.308	18.852	1	.000	3.807		

a. Variable(s) entered on step 1: M1_ca3, M5_ca7, F_SE, F_HL.

```
STATS OUTPUT ATTRS
ORIENTATION=PORTRAIT.
```
