# Supplementary material for: The effect of maternal decisional authority on children's vaccination in East Asia
Source: PLoS One. 2018 Jul 12;13(7):e0200333. doi: 10.1371/journal.pone.0200333 (PMC6042723; doi:10.1371/journal.pone.0200333)
Supplement: S2 Table — (PDF) [file pone.0200333.s006.pdf]

```
LOGISTIC REGRESSION VARIABLES Dep_4_bi
  /METHOD=ENTER M1_ca3 M5_ca7 F_SE F_HL B3 B3_n2 B3_n3 B3_n4 B3_n5
  /PRINT=CI(95)
  /CRITERIA=PIN(0.05) POUT(0.10) ITERATE(20) CUT(0.5).
```

Logistic Regression

Case Processing Summary

Unweighted Cases[a]		N	Percent
Selected Cases	Included in Analysis	1519	96.7
	Missing Cases	52	3.3
	Total	1571	100.0
Unselected Cases		0	.0
Total		1571	100.0

a. If weight is in effect, see classification table for the total number of cases.

Dependent Variable Encoding

Original Value	Internal Value
1가지 이상 미접종	0
모두 접종	1

Block 0: Beginning Block

Classification Table[a,b]

	Observed		Predicted		
			4가지 국가백신 접종여부		Percentage Correct
			1가지 이상 미접종	모두 접종	
Step 0	4가지 국가백신 접종여부	1가지 이상 미접종	0	266	.0
		모두 접종	0	1253	100.0
	Overall Percentage				82.5

a. Constant is included in the model.

b. The cut value is .500

Variables in the Equation

		B	S.E.	Wald	df	Sig.	Exp(B)
Step 0	Constant	1.550	.068	527.019	1	.000	4.711

Variables not in the Equation

			Score	df	Sig.
Step 0	Variables	M1_ca3	2.121	1	.145
		M5_ca7	.029	1	.865
		F_SE	3.414	1	.065
		F_HL	11.149	1	.001
		B3	.234	1	.629
		B3_n2	3.337	1	.068
		B3_n3	.437	1	.508
		B3_n4	.000	1	1.000
		B3_n5	2.018	1	.155
	Overall Statistics		24.125	9	.004

Block 1: Method = Enter

Omnibus Tests of Model Coefficients

		Chi-square	df	Sig.
Step 1	Step	23.906	9	.004
	Block	23.906	9	.004
	Model	23.906	9	.004

Model Summary

Step	-2 Log likelihood	Cox & Snell R Square	Nagelkerke R Square
1	1385.437[a]	.016	.026

a. Estimation terminated at iteration number 5 because parameter estimates changed by less than .001.

Classification Table[a]

Observed			Predicted		
			4가지 국가백신 접종여부		Percentage Correct
			1가지 이상 미접종	모두 접종	
Step 1	4가지 국가백신 접종여부	1가지 이상 미접종	0	266	.0
		모두 접종	0	1253	100.0
	Overall Percentage				82.5

a. The cut value is .500

Variables in the Equation

		B	S.E.	Wald	df	Sig.	Exp(B)	95% C.I.for EXP(B)	
								Lower	Upper
Step 1[a]	M1_ca3	.157	.153	1.053	1	.305	1.169	.867	1.577
	M5_ca7	-.017	.032	.267	1	.605	.984	.923	1.047
	F_SE	.116	.076	2.303	1	.129	1.123	.967	1.304
	F_HL	.194	.065	8.923	1	.003	1.214	1.069	1.379
	B3	.039	.114	.115	1	.735	1.039	.832	1.299
	B3_n2	.408	.202	4.069	1	.044	1.503	1.012	2.234
	B3_n3	-.053	.163	.105	1	.746	.948	.688	1.307
	B3_n4	-.127	.131	.947	1	.331	.880	.681	1.138
	B3_n5	-.177	.088	4.036	1	.045	.838	.705	.996
	Constant	1.504	.378	15.804	1	.000	4.498		

a. Variable(s) entered on step 1: M1_ca3, M5_ca7, F_SE, F_HL, B3, B3_n2, B3_n3, B3_n4, B3_n5.

```
USE ALL.
COMPUTE filter_$=(A5_2=1  &  C=1).
VARIABLE LABELS filter_$ 'A5_2=1  &  C=1 (FILTER)'.
VALUE LABELS filter_$ 0 'Not Selected' 1 'Selected'.
FORMATS filter_$ (f1.0).
FILTER BY filter_$.
EXECUTE.
LOGISTIC REGRESSION VARIABLES Dep_4_bi
  /METHOD=ENTER M1_ca3 M5_ca7 F_SE F_HL B3 B3_n2 B3_n3 B3_n4 B3_n5
  /PRINT=CI(95)
  /CRITERIA=PIN(0.05) POUT(0.10) ITERATE(20) CUT(0.5).
```

Logistic Regression

Case Processing Summary

Unweighted Cases[a]		N	Percent
Selected Cases	Included in Analysis	572	99.7
	Missing Cases	2	.3
	Total	574	100.0
Unselected Cases		0	.0
Total		574	100.0

a. If weight is in effect, see classification table for the total number of cases.

Dependent Variable Encoding

Original Value	Internal Value
1가지 이상 미접종	0
모두 접종	1

Block 0: Beginning Block

Classification Table[a,b]

Observed			Predicted		
			4가지 국가백신 접종여부		Percentage Correct
			1가지 이상 미접종	모두 접종	
Step 0	4가지 국가백신 접종여부	1가지 이상 미접종	0	52	.0
		모두 접종	0	520	100.0
	Overall Percentage				90.9

a. Constant is included in the model.

b. The cut value is .500

Variables in the Equation

		B	S.E.	Wald	df	Sig.	Exp(B)
Step 0	Constant	2.303	.145	250.635	1	.000	10.000

Variables not in the Equation

			Score	df	Sig.
Step 0	Variables	M1_ca3	.269	1	.604
		M5_ca7	.936	1	.333
		F_SE	.518	1	.472
		F_HL	.444	1	.505
		B3	1.009	1	.315
		B3_n2	.328	1	.567
		B3_n3	.125	1	.724
		B3_n4	.914	1	.339
		B3_n5	1.587	1	.208
	Overall Statistics		9.324	9	.408

Block 1: Method = Enter

Omnibus Tests of Model Coefficients

		Chi-square	df	Sig.
Step 1	Step	9.229	9	.416
	Block	9.229	9	.416
	Model	9.229	9	.416

Model Summary

Step	-2 Log likelihood	Cox & Snell R Square	Nagelkerke R Square
1	339.274[a]	.016	.035

a. Estimation terminated at iteration number 5 because parameter estimates changed by less than .001.

Classification Table[a]

Observed			Predicted		
			4가지 국가백신 접종여부		Percentage Correct
			1가지 이상 미접종	모두 접종	
Step 1	4가지 국가백신 접종여부	1가지 이상 미접종	0	52	.0
		모두 접종	0	520	100.0
	Overall Percentage				90.9

a. The cut value is .500

Variables in the Equation

		B	S.E.	Wald	df	Sig.	Exp(B)	95% C.I.for EXP(B) Lower	Upper
Step 1[a]	M1_ca3	-.191	.322	.354	1	.552	.826	.440	1.551
	M5_ca7	.077	.077	.999	1	.318	1.080	.929	1.257
	F_SE	.144	.175	.673	1	.412	1.155	.819	1.629
	F_HL	.089	.145	.375	1	.540	1.093	.822	1.453
	B3	.460	.255	3.269	1	.071	1.585	.962	2.610
	B3_n2	-.481	.440	1.193	1	.275	.618	.261	1.465
	B3_n3	.418	.451	.860	1	.354	1.519	.628	3.675
	B3_n4	-.286	.279	1.047	1	.306	.752	.435	1.299
	B3_n5	-.290	.192	2.276	1	.131	.748	.513	1.091
	Constant	2.933	.883	11.036	1	.001	18.776		

a. Variable(s) entered on step 1: M1_ca3, M5_ca7, F_SE, F_HL, B3, B3_n2, B3_n3, B3_n4, B3_n5.

```
USE ALL.
COMPUTE filter_$=(A5_2=1  &  C=2).
VARIABLE LABELS filter_$ 'A5_2=1  &  C=2 (FILTER)'.
```

```
VALUE LABELS filter_$ 0 'Not Selected' 1 'Selected'.
FORMATS filter_$ (f1.0).
FILTER BY filter_$.
EXECUTE.
LOGISTIC REGRESSION VARIABLES Dep_4_bi
  /METHOD=ENTER M1_ca3 M5_ca7 F_SE F_HL B3 B3_n2 B3_n3 B3_n4 B3_n5
  /PRINT=CI(95)
  /CRITERIA=PIN(0.05) POUT(0.10) ITERATE(20) CUT(0.5).
```

Logistic Regression

Case Processing Summary

Unweighted Cases[a]		N	Percent
Selected Cases	Included in Analysis	548	96.8
	Missing Cases	18	3.2
	Total	566	100.0
Unselected Cases		0	.0
Total		566	100.0

a. If weight is in effect, see classification table for the total number of cases.

Dependent Variable Encoding

Original Value	Internal Value
1가지 이상 미접종	0
모두 접종	1

Block 0: Beginning Block

Classification Table[a,b]

Observed			Predicted		
			4가지 국가백신 접종여부		Percentage Correct
			1가지 이상 미접종	모두 접종	
Step 0	4가지 국가백신 접종여부	1가지 이상 미접종	0	119	.0
		모두 접종	0	429	100.0
	Overall Percentage				78.3

a. Constant is included in the model.

b. The cut value is .500

Variables in the Equation

		B	S.E.	Wald	df	Sig.	Exp(B)
Step 0	Constant	1.282	.104	153.188	1	.000	3.605

Variables not in the Equation

			Score	df	Sig.
Step 0	Variables	M1_ca3	3.016	1	.082
		M5_ca7	1.156	1	.282
		F_SE	.963	1	.326
		F_HL	7.495	1	.006
		B3	.710	1	.399
		B3_n2	10.026	1	.002
		B3_n3	5.375	1	.020
		B3_n4	1.998	1	.157
		B3_n5	3.938	1	.047
	Overall Statistics		33.901	9	.000

Block 1: Method = Enter

Omnibus Tests of Model Coefficients

		Chi-square	df	Sig.
Step 1	Step	35.183	9	.000
	Block	35.183	9	.000
	Model	35.183	9	.000

Model Summary

Step	-2 Log likelihood	Cox & Snell R Square	Nagelkerke R Square
1	538.334[a]	.062	.096

a. Estimation terminated at iteration number 5 because parameter estimates changed by less than .001.

Classification Table[a]

Observed			Predicted: 4가지 국가백신 접종여부: 1가지 이상 미접종	Predicted: 4가지 국가백신 접종여부: 모두 접종	Predicted: Percentage Correct
Step 1	4가지 국가백신 접종여부	1가지 이상 미접종	2	117	1.7
		모두 접종	3	426	99.3
	Overall Percentage				78.1

a. The cut value is .500

Variables in the Equation

		B	S.E.	Wald	df	Sig.	Exp(B)	95% C.I.for EXP(B)	
								Lower	Upper
Step 1[a]	M1_ca3	.551	.358	2.365	1	.124	1.734	.860	3.499
	M5_ca7	-.012	.060	.038	1	.845	.988	.879	1.111
	F_SE	.056	.161	.121	1	.728	1.058	.771	1.450
	F_HL	.255	.124	4.220	1	.040	1.290	1.012	1.646
	B3	-.050	.170	.084	1	.771	.952	.681	1.329
	B3_n2	.469	.243	3.738	1	.053	1.599	.994	2.572
	B3_n3	.273	.205	1.764	1	.184	1.313	.878	1.964
	B3_n4	.167	.185	.807	1	.369	1.181	.821	1.699
	B3_n5	-.622	.171	13.275	1	.000	.537	.384	.750
	Constant	.571	.741	.595	1	.441	1.771		

a. Variable(s) entered on step 1: M1_ca3, M5_ca7, F_SE, F_HL, B3, B3_n2, B3_n3, B3_n4, B3_n5.

```
USE ALL.
COMPUTE filter_$=(A5_2=1  &  C=3).
VARIABLE LABELS filter_$ 'A5_2=1  &  C=3 (FILTER)'.
VALUE LABELS filter_$ 0 'Not Selected' 1 'Selected'.
FORMATS filter_$ (f1.0).
FILTER BY filter_$.
EXECUTE.
LOGISTIC REGRESSION VARIABLES Dep_4_bi
  /METHOD=ENTER M1_ca3 M5_ca7 F_SE F_HL B3 B3_n2 B3_n3 B3_n4 B3_n5
  /PRINT=CI(95)
  /CRITERIA=PIN(0.05) POUT(0.10) ITERATE(20) CUT(0.5).
```

Logistic Regression

Case Processing Summary

Unweighted Cases[a]		N	Percent
Selected Cases	Included in Analysis	399	92.6
	Missing Cases	32	7.4
	Total	431	100.0
Unselected Cases		0	.0
Total		431	100.0

a. If weight is in effect, see classification table for the total number of cases.

Dependent Variable Encoding

Original Value	Internal Value
1가지 이상 미접종	0
모두 접종	1

Block 0: Beginning Block

Classification Table[a,b]

Observed			Predicted		
			4가지 국가백신 접종여부		Percentage Correct
			1가지 이상 미접종	모두 접종	
Step 0	4가지 국가백신 접종여부	1가지 이상 미접종	0	95	.0
		모두 접종	0	304	100.0
	Overall Percentage				76.2

a. Constant is included in the model.

b. The cut value is .500

Variables in the Equation

		B	S.E.	Wald	df	Sig.	Exp(B)
Step 0	Constant	1.163	.118	97.926	1	.000	3.200

Variables not in the Equation

			Score	df	Sig.
Step 0	Variables	M1_ca3	.396	1	.529
		M5_ca7	.054	1	.815
		F_SE	.579	1	.447
		F_HL	9.243	1	.002
		B3	.839	1	.360
		B3_n2	2.843	1	.092
		B3_n3	.156	1	.693
		B3_n4	.358	1	.550
		B3_n5	2.693	1	.101
	Overall Statistics		18.585	9	.029

Block 1: Method = Enter

Omnibus Tests of Model Coefficients

		Chi-square	df	Sig.
Step 1	Step	20.594	9	.015
	Block	20.594	9	.015
	Model	20.594	9	.015

Model Summary

Step	-2 Log likelihood	Cox & Snell R Square	Nagelkerke R Square
1	417.408[a]	.050	.075

a. Estimation terminated at iteration number 20 because maximum iterations has been reached. Final solution cannot be found.

Classification Table[a]

			Predicted		
			4가지 국가백신 접종여부		Percentage Correct
Observed			1가지 이상 미접종	모두 접종	
Step 1	4가지 국가백신 접종여부	1가지 이상 미접종	7	88	7.4
		모두 접종	3	301	99.0
	Overall Percentage				77.2

a. The cut value is .500

Variables in the Equation

		B	S.E.	Wald	df	Sig.	Exp(B)	95% C.I.for EXP(B) Lower	95% C.I.for EXP(B) Upper
Step 1[a]	M1_ca3	.068	.233	.085	1	.771	1.070	.678	1.688
	M5_ca7	.005	.069	.006	1	.940	1.005	.878	1.151
	F_SE	.061	.134	.203	1	.652	1.062	.816	1.383
	F_HL	.306	.100	9.390	1	.002	1.358	1.117	1.652
	B3	-.170	.229	.556	1	.456	.843	.539	1.320
	B3_n2	19.480	9946.281	.000	1	.998	288430836.3	.000	.
	B3_n3	-.073	.546	.018	1	.894	.930	.319	2.712
	B3_n4	.286	.403	.503	1	.478	1.331	.604	2.930
	B3_n5	-.242	.142	2.887	1	.089	.785	.594	1.038
	Constant	-17.955	9946.281	.000	1	.999	.000		

a. Variable(s) entered on step 1: M1_ca3, M5_ca7, F_SE, F_HL, B3, B3_n2, B3_n3, B3_n4, B3_n5.

```
USE ALL.
COMPUTE filter_$=(A5_2=1 ).
VARIABLE LABELS filter_$ 'A5_2=1  (FILTER)'.
```

```
VALUE LABELS filter_$ 0 'Not Selected' 1 'Selected'.
FORMATS filter_$ (f1.0).
FILTER BY filter_$.
EXECUTE.
LOGISTIC REGRESSION VARIABLES Dep_4_bi
  /METHOD=ENTER M1_ca3 M5_ca7 F_SE F_HL
  /PRINT=CI(95)
  /CRITERIA=PIN(0.05) POUT(0.10) ITERATE(20) CUT(0.5).
```

Logistic Regression

Case Processing Summary

Unweighted Cases[a]		N	Percent
Selected Cases	Included in Analysis	1519	96.7
	Missing Cases	52	3.3
	Total	1571	100.0
Unselected Cases		0	.0
Total		1571	100.0

a. If weight is in effect, see classification table for the total number of cases.

Dependent Variable Encoding

Original Value	Internal Value
1가지 이상 미접종	0
모두 접종	1

Block 0: Beginning Block

Classification Table[a,b]

Observed			Predicted		
			4가지 국가백신 접종여부		Percentage Correct
			1가지 이상 미접종	모두 접종	
Step 0	4가지 국가백신 접종여부	1가지 이상 미접종	0	266	.0
		모두 접종	0	1253	100.0
	Overall Percentage				82.5

a. Constant is included in the model.

b. The cut value is .500

Variables in the Equation

		B	S.E.	Wald	df	Sig.	Exp(B)
Step 0	Constant	1.550	.068	527.019	1	.000	4.711

Variables not in the Equation

			Score	df	Sig.
Step 0	Variables	M1_ca3	2.121	1	.145
		M5_ca7	.029	1	.865
		F_SE	3.414	1	.065
		F_HL	11.149	1	.001
	Overall Statistics		13.828	4	.008

Block 1: Method = Enter

Omnibus Tests of Model Coefficients

		Chi-square	df	Sig.
Step 1	Step	13.098	4	.011
	Block	13.098	4	.011
	Model	13.098	4	.011

Model Summary

Step	-2 Log likelihood	Cox & Snell R Square	Nagelkerke R Square
1	1396.246[a]	.009	.014

a. Estimation terminated at iteration number 4 because parameter estimates changed by less than .001.

Classification Table[a]

Observed			Predicted		
			4가지 국가백신 접종여부		Percentage Correct
			1가지 이상 미접종	모두 접종	
Step 1	4가지 국가백신 접종여부	1가지 이상 미접종	0	266	.0
		모두 접종	0	1253	100.0
	Overall Percentage				82.5

a. The cut value is .500

Variables in the Equation

		B	S.E.	Wald	df	Sig.	Exp(B)	95% C.I.for EXP(B)	
								Lower	Upper
Step 1[a]	M1_ca3	.132	.151	.771	1	.380	1.142	.849	1.535
	M5_ca7	-.005	.032	.029	1	.865	.995	.935	1.058
	F_SE	.080	.069	1.369	1	.242	1.084	.947	1.240
	F_HL	.191	.064	8.834	1	.003	1.210	1.067	1.373
	Constant	1.337	.308	18.852	1	.000	3.807		

a. Variable(s) entered on step 1: M1_ca3, M5_ca7, F_SE, F_HL.

```
USE ALL.
COMPUTE filter_$=(A5_2=1  &  C=1).
VARIABLE LABELS filter_$ 'A5_2=1  &  C=1 (FILTER)'.
VALUE LABELS filter_$ 0 'Not Selected' 1 'Selected'.
FORMATS filter_$ (f1.0).
FILTER BY filter_$.
EXECUTE.
LOGISTIC REGRESSION VARIABLES Dep_4_bi
  /METHOD=ENTER M1_ca3 M5_ca7 F_SE F_HL
  /PRINT=CI(95)
  /CRITERIA=PIN(0.05) POUT(0.10) ITERATE(20) CUT(0.5).
```

Logistic Regression

Case Processing Summary

Unweighted Cases[a]		N	Percent
Selected Cases	Included in Analysis	572	99.7
	Missing Cases	2	.3
	Total	574	100.0
Unselected Cases		0	.0
Total		574	100.0

a. If weight is in effect, see classification table for the total number of cases.

Dependent Variable Encoding

Original Value	Internal Value
1가지 이상 미접종	0
모두 접종	1

Block 0: Beginning Block

Classification Table[a,b]

Observed			Predicted: 4가지 국가백신 접종여부: 1가지 이상 미접종	Predicted: 4가지 국가백신 접종여부: 모두 접종	Predicted: Percentage Correct
Step 0	4가지 국가백신 접종여부	1가지 이상 미접종	0	52	.0
		모두 접종	0	520	100.0
	Overall Percentage				90.9

a. Constant is included in the model.

b. The cut value is .500

Variables in the Equation

		B	S.E.	Wald	df	Sig.	Exp(B)
Step 0	Constant	2.303	.145	250.635	1	.000	10.000

Variables not in the Equation

			Score	df	Sig.
Step 0	Variables	M1_ca3	.269	1	.604
		M5_ca7	.936	1	.333
		F_SE	.518	1	.472
		F_HL	.444	1	.505
	Overall Statistics		2.232	4	.693

Block 1: Method = Enter

Omnibus Tests of Model Coefficients

		Chi-square	df	Sig.
Step 1	Step	2.234	4	.693
	Block	2.234	4	.693
	Model	2.234	4	.693

Model Summary

Step	-2 Log likelihood	Cox & Snell R Square	Nagelkerke R Square
1	346.269[a]	.004	.009

a. Estimation terminated at iteration number 5 because parameter estimates changed by less than .001.

Classification Table[a]

Observed			Predicted: 4가지 국가백신 접종여부		
			1가지 이상 미접종	모두 접종	Percentage Correct
Step 1	4가지 국가백신 접종여부	1가지 이상 미접종	0	52	.0
		모두 접종	0	520	100.0
	Overall Percentage				90.9

a. The cut value is .500

Variables in the Equation

		B	S.E.	Wald	df	Sig.	Exp(B)	95% C.I.for EXP(B) Lower	Upper
Step 1[a]	M1_ca3	-.275	.319	.745	1	.388	.760	.407	1.418
	M5_ca7	.074	.076	.931	1	.335	1.076	.927	1.250
	F_SE	.090	.167	.287	1	.592	1.094	.788	1.518
	F_HL	.081	.142	.323	1	.570	1.084	.821	1.432
	Constant	2.566	.645	15.854	1	.000	13.020		

a. Variable(s) entered on step 1: M1_ca3, M5_ca7, F_SE, F_HL.

```
USE ALL.
COMPUTE filter_$=(A5_2=1  &  C=2).
VARIABLE LABELS filter_$ 'A5_2=1  &  C=2 (FILTER)'.
VALUE LABELS filter_$ 0 'Not Selected' 1 'Selected'.
FORMATS filter_$ (f1.0).
FILTER BY filter_$.
EXECUTE.
LOGISTIC REGRESSION VARIABLES Dep_4_bi
  /METHOD=ENTER M1_ca3 M5_ca7 F_SE F_HL
  /PRINT=CI(95)
  /CRITERIA=PIN(0.05) POUT(0.10) ITERATE(20) CUT(0.5).
```

Logistic Regression

Case Processing Summary

Unweighted Cases[a]		N	Percent
Selected Cases	Included in Analysis	548	96.8
	Missing Cases	18	3.2
	Total	566	100.0
Unselected Cases		0	.0
Total		566	100.0

a. If weight is in effect, see classification table for the total number of cases.

Dependent Variable Encoding

Original Value	Internal Value
1가지 이상 미접종	0
모두 접종	1

Block 0: Beginning Block

Classification Table[a,b]

Observed			Predicted		
			4가지 국가백신 접종여부		Percentage Correct
			1가지 이상 미접종	모두 접종	
Step 0	4가지 국가백신 접종여부	1가지 이상 미접종	0	119	.0
		모두 접종	0	429	100.0
	Overall Percentage				78.3

a. Constant is included in the model.

b. The cut value is .500

Variables in the Equation

		B	S.E.	Wald	df	Sig.	Exp(B)
Step 0	Constant	1.282	.104	153.188	1	.000	3.605

Variables not in the Equation

			Score	df	Sig.
Step 0	Variables	M1_ca3	3.016	1	.082
		M5_ca7	1.156	1	.282
		F_SE	.963	1	.326
		F_HL	7.495	1	.006
	Overall Statistics		10.000	4	.040

Block 1: Method = Enter

Omnibus Tests of Model Coefficients

		Chi-square	df	Sig.
Step 1	Step	9.461	4	.051
	Block	9.461	4	.051
	Model	9.461	4	.051

Model Summary

Step	-2 Log likelihood	Cox & Snell R Square	Nagelkerke R Square
1	564.056[a]	.017	.026

a. Estimation terminated at iteration number 4 because parameter estimates changed by less than .001.

Classification Table[a]

Observed			Predicted		
			4가지 국가백신 접종여부		Percentage Correct
			1가지 이상 미접종	모두 접종	
Step 1	4가지 국가백신 접종여부	1가지 이상 미접종	0	119	.0
		모두 접종	1	428	99.8
	Overall Percentage				78.1

a. The cut value is .500

Variables in the Equation

		B	S.E.	Wald	df	Sig.	Exp(B)	95% C.I.for EXP(B) Lower	95% C.I.for EXP(B) Upper
Step 1[a]	M1_ca3	.360	.345	1.089	1	.297	1.433	.729	2.818
	M5_ca7	.040	.053	.586	1	.444	1.041	.939	1.154
	F_SE	.049	.154	.102	1	.749	1.050	.777	1.419
	F_HL	.286	.120	5.710	1	.017	1.331	1.053	1.683
	Constant	.386	.683	.320	1	.572	1.471		

a. Variable(s) entered on step 1: M1_ca3, M5_ca7, F_SE, F_HL.

```
USE ALL.
COMPUTE filter_$=(A5_2=1  &  C=3).
VARIABLE LABELS filter_$ 'A5_2=1  &  C=3 (FILTER)'.
VALUE LABELS filter_$ 0 'Not Selected' 1 'Selected'.
FORMATS filter_$ (f1.0).
FILTER BY filter_$.
```

```
EXECUTE.
LOGISTIC REGRESSION VARIABLES Dep_4_bi
  /METHOD=ENTER M1_ca3 M5_ca7 F_SE F_HL
  /PRINT=CI(95)
  /CRITERIA=PIN(0.05) POUT(0.10) ITERATE(20) CUT(0.5).
```

Logistic Regression

Case Processing Summary

Unweighted Cases[a]		N	Percent
Selected Cases	Included in Analysis	399	92.6
	Missing Cases	32	7.4
	Total	431	100.0
Unselected Cases		0	.0
Total		431	100.0

a. If weight is in effect, see classification table for the total number of cases.

Dependent Variable Encoding

Original Value	Internal Value
1가지 이상 미접종	0
모두 접종	1

Block 0: Beginning Block

Classification Table[a,b]

Observed			Predicted		
			4가지 국가백신 접종여부		Percentage Correct
			1가지 이상 미접종	모두 접종	
Step 0	4가지 국가백신 접종여부	1가지 이상 미접종	0	95	.0
		모두 접종	0	304	100.0
	Overall Percentage				76.2

a. Constant is included in the model.

b. The cut value is .500

Variables in the Equation

		B	S.E.	Wald	df	Sig.	Exp(B)
Step 0	Constant	1.163	.118	97.926	1	.000	3.200

Variables not in the Equation

			Score	df	Sig.
Step 0	Variables	M1_ca3	.396	1	.529
		M5_ca7	.054	1	.815
		F_SE	.579	1	.447
		F_HL	9.243	1	.002
	Overall Statistics		9.448	4	.051

Block 1: Method = Enter

Omnibus Tests of Model Coefficients

		Chi-square	df	Sig.
Step 1	Step	8.788	4	.067
	Block	8.788	4	.067
	Model	8.788	4	.067

Model Summary

Step	-2 Log likelihood	Cox & Snell R Square	Nagelkerke R Square
1	429.213[a]	.022	.033

a. Estimation terminated at iteration number 4 because parameter estimates changed by less than .001.

Classification Table[a]

Observed			Predicted		
			4가지 국가백신 접종여부		Percentage Correct
			1가지 이상 미접종	모두 접종	
Step 1	4가지 국가백신 접종여부	1가지 이상 미접종	2	93	2.1
		모두 접종	1	303	99.7
	Overall Percentage				76.4

a. The cut value is .500

Variables in the Equation

		B	S.E.	Wald	df	Sig.	Exp(B)	95% C.I.for EXP(B)	
								Lower	Upper
Step 1[a]	M1_ca3	.102	.227	.201	1	.654	1.107	.710	1.727
	M5_ca7	-.014	.068	.045	1	.833	.986	.862	1.127
	F_SE	.014	.130	.012	1	.914	1.014	.786	1.309
	F_HL	.277	.097	8.109	1	.004	1.320	1.090	1.597
	Constant	1.104	.507	4.751	1	.029	3.016		

a. Variable(s) entered on step 1: M1_ca3, M5_ca7, F_SE, F_HL.

```
CORRELATIONS
  /VARIABLES=Dep_4_bi M1_ca3 M5_ca7 F_MC1 F_MC2 F_HL F_ISB F_SE
  /PRINT=TWOTAIL NOSIG
  /MISSING=PAIRWISE.
```

Correlations

Correlations

		4가지 국가백신 접종여부	교육수준(3)	소득수준(7)	모성역량(1) 팩터	모성역량(2) 팩터	건강문해력 팩터	건강정보탐색 B3 팩터	자기효능감F2 (13568) 팩터
4가지 국가백신 접종여부	Pearson Correlation	1	.031	.012	.109*	.059	.152**	-.048	.038
	Sig. (2-tailed)		.531	.816	.030	.243	.002	.336	.448
	N	399	399	399	398	399	399	399	399
교육수준(3)	Pearson Correlation	.031	1	.253**	-.053	.087	.057	.013	.087
	Sig. (2-tailed)	.531		.000	.270	.071	.241	.783	.070
	N	399	431	431	430	431	431	431	431
소득수준(7)	Pearson Correlation	.012	.253**	1	.107*	.193**	.129**	.039	.224**
	Sig. (2-tailed)	.816	.000		.027	.000	.007	.423	.000
	N	399	431	431	430	431	431	431	431
모성역량(1) 팩터	Pearson Correlation	.109*	-.053	.107*	1	.098*	.117*	.082	.178**
	Sig. (2-tailed)	.030	.270	.027		.041	.015	.088	.000
	N	398	430	430	430	430	430	430	430
모성역량(2) 팩터	Pearson Correlation	.059	.087	.193**	.098*	1	.131**	.205**	.490**
	Sig. (2-tailed)	.243	.071	.000	.041		.007	.000	.000
	N	399	431	431	430	431	431	431	431
건강문해력 팩터	Pearson Correlation	.152**	.057	.129**	.117*	.131**	1	-.006	.189**
	Sig. (2-tailed)	.002	.241	.007	.015	.007		.901	.000
	N	399	431	431	430	431	431	431	431
건강정보탐색B3 팩터	Pearson Correlation	-.048	.013	.039	.082	.205**	-.006	1	.162**
	Sig. (2-tailed)	.336	.783	.423	.088	.000	.901		.001
	N	399	431	431	430	431	431	431	431
자기효능감F2(13568) 팩터	Pearson Correlation	.038	.087	.224**	.178**	.490**	.189**	.162**	1
	Sig. (2-tailed)	.448	.070	.000	.000	.000	.000	.001	
	N	399	431	431	430	431	431	431	431

*. Correlation is significant at the 0.05 level (2-tailed).

**. Correlation is significant at the 0.01 level (2-tailed).

```
USE ALL.
COMPUTE filter_$=(A5_2=1 ).
VARIABLE LABELS filter_$ 'A5_2=1  (FILTER)'.
VALUE LABELS filter_$ 0 'Not Selected' 1 'Selected'.
FORMATS filter_$ (f1.0).
FILTER BY filter_$.
EXECUTE.
```
